# The neural characteristics influencing literacy outcome in children with cochlear implants

**DOI:** 10.1093/braincomms/fcaf086

**Published:** 2025-02-21

**Authors:** Nabin Koirala, Jacy Manning, Sara Neumann, Chelsea Anderson, Mickael L D Deroche, Jace Wolfe, Kenneth Pugh, Nicole Landi, Muthuraman Muthuraman, Vincent L Gracco

**Affiliations:** Child Study Center, School of Medicine, Yale University, New Haven, CT 06511, USA; Brain Imaging Research Core, University of Connecticut, Storrs, CT 06269, USA; Nathan Kline Institute for Psychiatric Research, Orangeburg, NY 10962, USA; Hearts for Hearing Foundation, Oklahoma City, OK 73120, USA; Hearts for Hearing Foundation, Oklahoma City, OK 73120, USA; Hearts for Hearing Foundation, Oklahoma City, OK 73120, USA; Department of Psychology, Concordia University, Montreal, QC H3G 1M8, Canada; Oberkotter Foundation, Philadelphia, PA 19102, USA; Child Study Center, School of Medicine, Yale University, New Haven, CT 06511, USA; Department of Psychological Sciences, University of Connecticut, Storrs, CT 06269, USA; Child Study Center, School of Medicine, Yale University, New Haven, CT 06511, USA; Department of Psychological Sciences, University of Connecticut, Storrs, CT 06269, USA; Department of Neurology, Universitätsklinikum Würzburg, Würzburg 97080, Germany; Child Study Center, School of Medicine, Yale University, New Haven, CT 06511, USA; School of Communication Sciences and Disorders, McGill University, Montreal, QC H3A 0G4, Canada

**Keywords:** cochlear implant, neural adaptation, reading, development

## Abstract

Early hearing intervention in children with congenital hearing loss is critical for improving auditory development, speech recognition and both expressive and receptive language, which translates into better educational outcomes and quality of life. In children receiving hearing aids or cochlear implants, both adaptive and potentially maladaptive neural reorganization can mitigate higher-level functions that impact reading. The focus of the present study was to dissect the neural underpinnings of the reading networks in children with cochlear implants and assess how these networks mediate the reading ability in children with cochlear implants. Cortical activity was obtained using naturalistic stimuli from 75 children (50 cochlear implant recipients, aged 7–17, and 25 age-matched children with typical hearing) using functional near-infrared spectroscopy. Assessment of basic reading skill was completed using the Reading Inventory and Scholastic Evaluation. We computed directed functional connectivity of the haemodynamic activity in reading-associated anterior and posterior brain regions using the time–frequency causality estimation method known as temporal partial directed coherence. The influence of the cochlear implant-related clinical measures on reading outcome and the extent to which neural connectivity mediated these effects were examined using structural equation modelling. Our findings reveal that the timing of intervention (e.g. age of first cochlear implants, age of first hearing aid) in children with cochlear implants significantly influenced their reading ability. Furthermore, reading-related processes (word recognition and decoding, vocabulary, morphology and sentence processing) were substantially mediated by the directed functional connectivity within reading-related neural circuits. Notably, the impact of these effects differed across various reading skills. Hearing age, defined as the age at which a participant received adequate access to sound, and age of initial implantation emerged as robust predictors of reading proficiency. The current study is one of the first to identify the influence of neural characteristics on reading outcomes for children with cochlear implants. The findings emphasize the importance of the duration of deafness and early intervention for enhancing outcomes and strengthening neural network connections. However, the neural evidence further suggested that such positive influences cannot fully offset the detrimental impact of early auditory deprivation. Consequently, additional, perhaps more specialized, interventions might be necessary to maximize the benefits of early prosthetic hearing intervention.

## Teaser

Koirala *et al*. report how neural underpinnings of the reading network are mediated in children with cochlear implants, highlighting the importance of intervention timing and duration of deafness. Their study shows early auditory deprivation poses persistent challenges to optimal reading development, underscoring the need for specialized support.

## Graphical Abstract

**Graphical Abstract:**
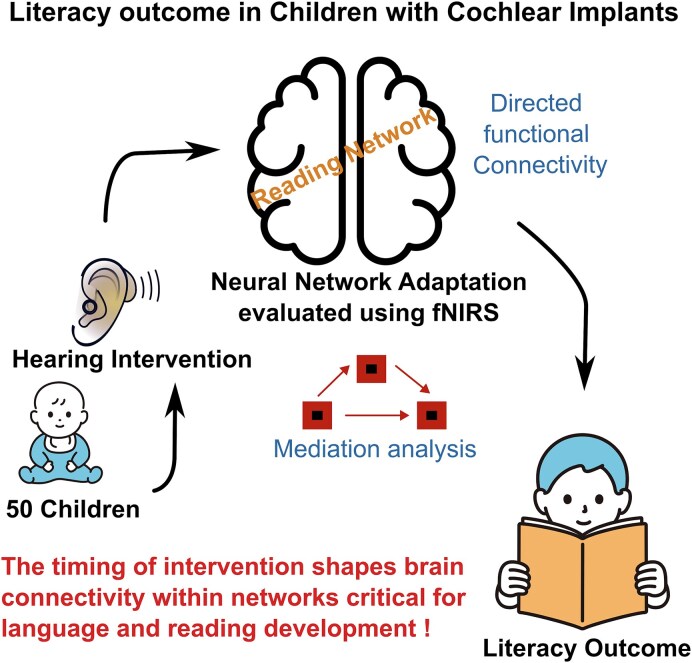

## Introduction

After sensory loss due to deafness, there are significant changes in neural organization that impact neurodevelopment.^1-4^ In children with congenital hearing loss, structural changes consistent with a delay in myelination and microstructural alteration,^5,6^ reduced spontaneous haemodynamic fluctuations and reduced connectivity in resting state have been reported for auditory, language and cognitive-related brain areas.^7,8^ During development, major fibre pathways connecting brain regions provide the scaffold for long-range connections and large-scale neural system interactions.^9-11^ Neuroimaging prior to cochlear implantation has been instrumental in identifying brain markers that forecast speech and language outcomes postimplantation. For example, the density of grey matter and white matter in the brain regions that remain unaffected by auditory deprivation before receiving a cochlear implant (CI) were the best predictors of speech perception postimplantation.^12^ Additionally, increased preimplant functional activation in the temporo-parieto-occipital junction and prefrontal cortex has been associated with postimplant language performance.^13^ These findings highlight the potential of brain imaging to predict rehabilitation success with cochlear prostheses.

Early sensory deprivation linked changes in brain structure and function can negatively impact neurodevelopmental scaffolding necessary for language and reading skills. For children born deaf or with severe hearing loss, speech and language delays are common along with subsequent challenges to reading and cognitive development.^14-18^ Newborn hearing screening, earlier age of prosthetic intervention to restore hearing, and increased availability of auditory and/or language-based therapy have collectively had a positive impact on subsequent language and reading development^19-29^ especially if the interventions occur before the closing of neurodevelopmental plasticity.^30-33^ Beyond the importance of language for communication and subsequent cognitive development, skilled reading requires children to build a functional neurocircuitry for printed-language processing that integrates with spoken-language-processing networks in typically hearing (TH) children.^34-36^ Previous studies have established that this functional neurocircuitry primarily involves two complementary circuits or streams; a dorsal stream and a ventral stream, which include occipitotemporal (ventral/dorsal), temporal (ventral), temporoparietal (dorsal) and anterior frontal (dorsal) brain areas.^37-42^ For children with hearing loss, a lack of input from the cochlea impacts connectivity within the auditory pathway, the primary auditory cortex, the higher-order auditory cortical regions, the multisensory cortex and subsequent connections with supramodal (heteromodal) processing regions.^43,44^ While earlier access to hearing is important to the development of speech and language,^1,2,28,45-48^ it is not clear how early access impacts the developing connectome.^49,50^

There is an extensive literature on the importance of early intervention (before 18 months of age) and the behavioural consequences of the intervention, specific details on the neural impact of intervention timing on the neural connectivity are limited. Research on the brain morphological changes in children with hearing loss suggests that congenital sensory deprivation creates a developmental delay in the myelination of the auditory neural pathway^51-53^ (cf. Simon *et al.*^54^ for review) and these changes are negatively correlated with categories of auditory performance (CAP) scores 12 months post implantation.^53^ Machine learning and predictive models based on data from voxel-based morphometry and multivoxel pattern similarity analyses have predicted individual children’s speech recognition at 6 months post-CI activation based on the neural preservation of higher-level auditory regions that were unaffected by the early hearing deprivation.^12^ These findings in relation to outcomes do reveal how differences in intervention timing impact the neural functioning for later developing language and reading. Additionally, while behavioural intervention has had a positive impact on language development, subsequent assessments have noted a regression of expressive and receptive language skills suggesting a weak neural foundation for the development of more complex language skills.^55^ School-age children with CI often display reductions in phonological awareness, vocabulary, decoding and reading comprehension compared with their TH peers.^39,56^ Overall, for children with CI, the effects of early hearing loss present continual challenges in the age-appropriate reading development.

In the current study, we explored whether and how hearing intervention factors (timing and kind of hearing augmentation) and development influence reading outcomes in school-aged children with CI, focusing on the mediating role of neural connectivity in the regions that support language and reading development. To assess neural connectivity, we recorded functional near-infrared spectroscopy (fNIRS) data, a technique well suited for characterizing brain network connectivity in children with CI (cf. Cai *et al.*^57^ and Lin *et al.*^58^) using naturalistic stimuli to ensure a controlled rest condition. The brain activity was recorded while watching a 7 min computer-generated animation (Inscapes^59^) that was created to watch during research brain scans. The video provides enough stimulation to keep children engaged while minimizing certain aspects of cognitive processing. Compared to traditional resting-state paradigms, Inscapes maintains similar functional connectivity patterns, particularly in the default network, while improving participant compliance.^59,60^ Overall, network connectivity captured at rest or under controlled resting conditions reflects a number of functional, task-related networks and has been used to identify biomarkers of brain functioning in multiple clinical populations^61-64^ and in large-scale longitudinal studies.^65-67^ In the present study, we utilized Inscapes to maintain compliance across the age range of the cohorts and relied on a sampling of reading fundamentals to assess how connectome changes were associated with reading ability. We assessed the relationship between neural connectivity strength to various aspects of reading measures and tested how the initial hearing intervention impacted reading-related neural connectivity. Further, we examined the effects of development using chronological age and hearing age (defined as the age at which a participant received adequate access to sound, computed as chronological age minus the duration of deafness) on network connectivity and reading ability to gauge the effects of early deafness. Overall, we were able to identify the neural consequences of early deafness on reading ability and its development in children with CI.

## Materials and methods

### Participants

A total of 75 children participated in the study of which 50 children (ages 7–17 years) had congenital bilateral severe-to-profound hearing loss and wore CIs and 25 were TH children. Inclusion criteria were: At least one CI by 4 years of age; primary communication via listening and spoken language in American English (i.e. limited use of sign language in most daily listening settings); minimum of 6 h of CI use per day as indicated by data logging or parent report. Exclusion criteria: no additional disabilities that could induce delays in language development and no anatomical abnormalities that could cause delays in language development such as ossification after bacterial meningitis, cochlear nerve deficiency or significant cochlear deformities (additional details on demographics, therapy attendance and speech recognition performance is reported in our previous study^20,68^). All implanted children had similar aided thresholds, ∼20–30 dB at frequencies between 250 and 6000 Hz, implying that they were all properly fitted. Forty-five children were bilaterally implanted with the second implantation occurring ∼16–17 months after the first implant.

### Experiment setup and data acquisition

**Figure 1:**
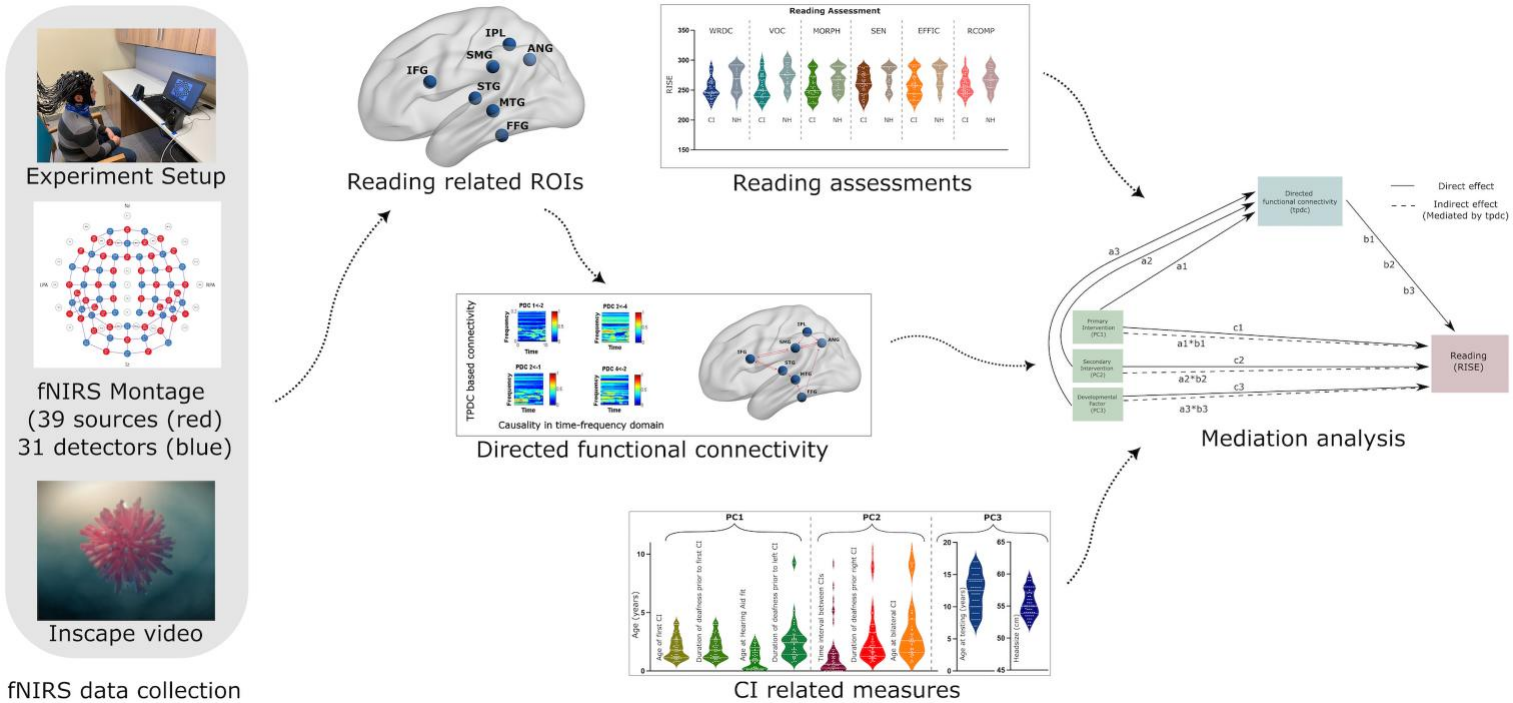
**Methodological pipeline.** The data acquisition and analysis steps used in the study.

Brain activity was collected using fNIRS from 122 channels and was recorded using 39 LED sources and 31 detectors from the NIRScout system developed by *NIRx Medical Technologies LLC* (https://nirx.net/) (see theoretical montage as shown in Fig. 1**)**. Each source emits near-infrared light at two wavelengths: 760 and 850 nm. An EasyCap (EASYCAP GmbH, Germany) was used to secure the sources and detectors, and their positions were registered using the Structure Sensor Pro application by Occipital Inc. (https://structure.io/structure-sensor-pro) with three fiducials (nasion and left/right preauricular point) and later digitized using the FieldTrip Matlab toolbox.^69^ The experiment was conducted in a dimly lit and quiet (e.g. background noise measured at 35dBA or less) room with participants seated comfortably upright in an adjustable chair. Participants were instructed to remain relaxed and to refrain from excessive movement. After positioning fNIRS probes over the head, fNIRS data recording were initiated, with a 7 min recording while the subjects were watching Inscapes—a movie paradigm that features abstract shapes without a narrative or scene-cuts.^59^ During the acquisition, the sound processors were off for the children with CI.

#### Clinical measures

We acquired several clinically relevant information on the participants to assess the impact of intervention timing and overall development. The following variables were obtained for each child with a CI: age of first CI implant, age of first hearing aid fitting, duration of deafness prior to first CI, duration of deafness prior to left and right CI, time between bilateral implantation, age of bilateral implantation, age of testing and head size.

#### Behavioural measures

All participants completed a battery of audio and speech tests using the Pediatric Minimum Speech Test Battery (PMSTB) protocol^70^ and consonant–nucleus–consonant (CNC) test^71^ along with standardized assessments of language and reading skills. Speech recognition scores for the CI group were recently reported and differed substantially from those of typical hearing (TH) children.^20^ Each child’s reading ability was assessed using the third edition of the Reading Inventory and Scholastic Evaluation (RISE), a 45–60 min computer-administered reading components assessment comprising six subtests specifically designed for grades 3–12. The six subtests include: word recognition and decoding (WRDC); vocabulary (VOC); morphology (MORPH); sentence processing (SEN); efficiency of basic reading comprehension (EFFIC) and reading comprehension (RCOMP), each of which targets a specific or foundational subskill related to reading comprehension performance. The subtests are modelled on the kinds of academic materials (words, sentences and passages) that are encountered in school curricula and designed to target a wider range of below-grade-level at-risk readers.^72^ The subtests are moderately to strongly correlated with each other indicating that they are measuring distinct yet overlapping aspects of reading ability.^72^ The RISE implements an item response theory (IRT) framework to facilitate result comparison across different test form. In this approach, each of the six subtests is scaled to have a mean of 250 and SD of 15, while adhering to a scale constraint with a minimum value of 190 and maximum value of 310 for each grade level ranging from 3rd to 12th grade.^72^

All experimental procedures were approved by the Institutional Review Board (IRB) of the data collection site (Hearts for Hearing) and subjects’ written informed consent was obtained from their legal guardian. Each participant was compensated financially for their participation and the experiment was conducted with the ethical guidelines as outlined in the declaration of Helsinki and was approved by the Western Institutional Review Board (reference #20190882). An illustration of the methodological pipeline is presented in Fig. 1.

### Data processing

#### Preprocessing

All obtained data were processed offline. The raw data (light intensity) were extracted from the acquisition software *Aurora fNIRS* and a MATLAB-based (MathWorks Inc., USA) in-house script, based on HomER^73^ that converts the raw data into optical density (OD),^74^ where the change in light observation referred as delta optical density (ΔOD) was calculated from the normalized changes in light incident on a detector from a source position defined by the equation


$${\Delta \mathrm{OD}}_{ij(t)}^{\lambda }=Ln\left[\frac{\Phi_{ij(0)}^{\lambda }}{\Phi_{ij(t)}^{\lambda }}\right]$$

where Φ is the intensity, *i* is the source position, *j* is the detector position and *λ* the wavelength of light. Any motion artefacts were removed by applying the moving standard deviation and spline interpolation methods,^75^ followed by the wavelet artefact correction^76^ (with probability threshold *α* = 0.1).^77^ To access functionally evoked changes in cortical oxyhaemoglobin (HbO) and deoxyhaemoglobin (HbR) relative concentrations (expressed in µM), the modified Beer–Lambert law^78^ was applied to the OD data that includes an age-dependent constant differential path length factor (4.99 + 0.067 × Age^0.814^).^79^ A linear detrending was used to remove slow drifts, and the time series is centred to zero mean to satisfy the criteria of second-order stationarity. Importantly, we did not use any filtering on the data before further analysis, as it has been previously shown that it could lead to spurious connections.^80^

The primary focus on the project was to determine whether the reading scores of children with CI were driven by differences in connectivity within the canonical reading network. Based on previous literature suggesting that differences in laterality are related to aspects of language representation in children with CI, we selected seven previously established bilateral language and reading-related brain regions. This includes the inferior frontal gyrus (IFG), superior temporal gyrus (STG), medial temporal gyrus (MTG), fusiform gyrus (FFG), supramarginal gyrus (SMG), inferior parietal lobule (IPL) and angular gyrus (ANG) in both hemispheres.^37-40^ It is important to note that the findings in this study are not hemisphere-specific but rather pertain to the regions of interest (ROIs).

After positioning the head cap on the vertex location (Cz), of the 122 recorded channels, 40 were selected and pooled into ROIs based on anatomical relevance to the language network (8 for IFG, 6 For STG, 8 for MTG, 6 for FFG, 4 for SMG, 4 for IPL and 4 for AG). The NFRI function^81^ was used to extract the Montreal Neurological Institute coordinates (MNI).

#### Directed functional connectivity analysis

The effective connectivity or directed functional connectivity analysis for both left and right probes was averaged and the time–frequency causality estimation method of temporal partial directed coherence (TPDC) based on dual-extended Kalman filtering^82,83^ was used to estimate the time-dependent autoregressive coefficients for each ROI. For nonlinear signals like fNIRS, the model is time varying to estimate the coefficients regularly over the course of the time-period, a process termed as adaptive autoregressive process and can be computed as


$$x(t)\;=\;\overset{r=p}{\underset{r=1}{\sum }}\;a_{r}(t)x(t-r)+\;\eta (t)$$

where *a_r_*(*t*) is the time-varying multivariate auto-regressive (MVAR) coefficients, *P* is the model order of time series *x*(*t*) and *η*(*t*) is the zero-mean Gaussian noise process. The extended Kalman filter used in the TPDC analysis is a predictor–corrector algorithm that estimates the states of a process, i.e. one extended Kalman filter estimates the states and feeds this information to a subsequent Kalman filter that estimates the model parameters and shares this information with the previous estimate. Hence, by using two Kalman filters in parallel, we were able to estimate the states and model parameters of the system at each time instant. The causality at each instant can be computed as the partial directed coherence (PDC) using MVAR autoregressive coefficients. An fNIRS time series can be modelled using a general nonlinear state-space model where at each time point, previous state estimates and weight estimates are fed to both Kalman filters. Both predictors are then corrected based on observed data, such that they yield current state and weight estimates. The time-dependent MVAR coefficients were estimated and used to compute the causality within the time series (PDC) by using the Granger causality^74^ as


$$|\pi_{i\leftarrow j}(\lambda )|=\;\frac{\left|A_{ij}(\lambda )\right|}{\sqrt{\sum k{\left|A_{kj}(\lambda )\right|}^{2}}}$$

where *A_ij_*(*λ*) is the *i* and *j*th element of *A*(*λ*) and the PDC is normalized such that $0\leq |\pi_{i\leftarrow j}(\lambda ){|}^{2}\leq 1$. Hence by calculating the PDC at each time point, multiple matrices corresponding to the time–frequency causality from the time series are obtained. As short channels were not included in our montage, the nonneural components were removed using the spatial domain regression filtering methods which have been shown to remove similar nonneural components as using the short channels represented in scalp haemodynamics from fNIRS signals^84^ which has been validated in our previous studies in both task-based and resting-state fNIRS data.^85,86^ Additionally, for the fNIRS time series, the frequency band of 0.009–0.08 Hz was extracted which has been previously shown to reflect the neurovascular coupling^87,88^ and avoids the Mayer waves frequency (∼0.1 Hz) which is shown to induce spurious correlations in connectivity measures for resting state data.^89^

#### Statistical analysis

For establishing the statistical significance of TPDC values, the stationarity of the time series is tested using an augmented Dickey–Fuller test.^90^ The TPDC measures have a nonlinear relation to the time series data from which they are derived, hence testing for significance cannot be performed using the standard tests for significance. A bootstrapping method as detailed in Kaminski *et al.*^91^ and Muthuraman *et al*.^92^ was applied for determining the significance. Using this method, for each participant, the original time series of length *N* was divided into *K* smaller nonoverlapping windows of length *V*, so that *N* = *KV*. The order of these windows was randomly shuffled to create a bootstrapped sample of the original time series. Then, a multivariate autoregressive model is fitted to the shuffled time series, and the TPDC values are estimated. The process is performed for 1000 iterations for each connection separately and the 99th percentile of the shuffled TPDC values (tail end of the distribution) is then considered as the threshold for significance for the actual TPDC value. This ensures that only the strongest TPDC values from the real data, which exceed this threshold, are considered significant. To note, this process is performed separately for each subject at each time point and for all frequency bands (please refer to Vergotte *et al*.^74^ for more details and flowchart in Supplementary Fig. 1). An open-source MATLAB package autoregressive fit (ARFIT)^93^ was used for estimating the autoregressive coefficients from the spatially filtered source signals. Finally, as volume conduction severely limits the neurophysiological interpretability of sensor-space connectivity analyses,^94,95^ the reliability of the connectivity was assessed using the time-reversal technique (TRT).^96^ TRT was applied as a second significance test on the connections identified by TPDC using bootstrapping algorithm. Hence, for each of the seven ROIs, we assessed connectivity to all other six regions, resulting in 42 unique connections for each RISE subtest (7 ROIs × 6 possible connections = 42). With six RISE subtests, this leads to a total of 252 possible connections (42 connections per subtest × 6 subtests = 252).

#### Mediation analysis

Mediation analysis was used to assess the influence of connectivity measures in the reading networks on the reading outcomes. The mediation analysis was performed using structural equation modelling (SEM)^97^ as implemented in the lavaan package in R (https://cran.r-project.org/web/packages/lavaan/index.html) for latent variable analysis. For this analysis, the predictor variables included CI-related clinical measures (age of first CI implant, age of first hearing aid fitting, duration of deafness prior to first CI, duration of deafness prior to left and right CI, time between bilateral implantation, age of bilateral implantation) and age of testing and head size, the mediating variables were the TPDC derived connectivity profile of the selected brain regions, and the outcome measures were the reading assessments of the children measured using the RISE subtests. Each mediation analysis was conducted separately to test whether the connectivity significantly mediated the influence of the principal components. The obtained *P*-values were corrected for multiple comparison corrections using Benjamini–Hochberg (BH) procedure.

#### Principal components analysis

In order to evaluate the influence of the clinical measures on the connectivity measures, we subjected the 10 clinical measures as listed above to a principal component analysis (PCA).^98^ The resulting components were then used to examine their effects individually for each of the outcome variables (RISE subtests).

## Results

### Sample characteristics

There was no significant difference between the sex, chronological age or hearing age [HA = chronological age—age of first hearing intervention (either CI or hearing aid)] of the children with CI when compared with TH children (Table 1).

**Table 1:** Demographics details and descriptive statistics for the sample used in the study

Group statistics					Welch’s *t*-test		
Variable	Group	*N* (females)	Mean (years)	SD	*t*-stats	df	*P*-value
Age	NH	25 (13)	12.08	3.10805	−0.188	45.267	0.852
	CI	50 (25)	12.22	2.90172			
Hearing age	NH	25 (13)	12.08	3.10805	1.161	44.61	0.252
	CI	50 (25)	11.2194	2.85341			
Sex	NH	25 (13)			−0.161	47.701	0.873
	CI	50 (25)					
Age at hearing aid fit	CI		1.0006	0.8507			
Age at first CI	CI		1.9832	0.9751			
Age at bilateral CI	CI		3.4152	2.568			
Ethnicity	NH	1 African American, 23 Caucasian, 1 Hispanic					
	CI	3 African American, 2 Asian, 34 Caucasian, 5 Hispanic, 2 Mixed Race, 4 Native American					

**Figure 2:**
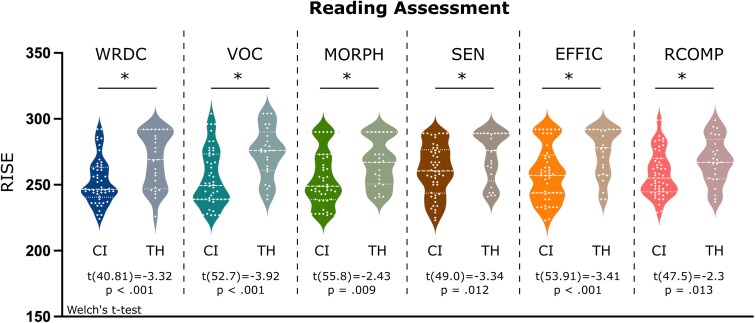
**Reading assessment.** Distribution of RISE scores for each subtest separately for children with CI and TH children. WRDC, word recognition and decoding; VOC, vocabulary; MORPH, morphology; SEN, sentence processing; EFFIC, efficiency of basic reading comprehension; RCOMP, reading comprehension.

The reading assessment scores (subtests of RISE) were significantly greater (*P* < 0.05) for TH children compared with the children with CI (Fig. 2). The mean scores (tested using Welch’s *t*-test), as well as the distribution (tested using the Mann–Whitney U-test), were significantly different between the groups. Specifically, RISE: WRDC [Welch’s *t*-test: *t*(40.81) = −3.32, *P* < 0.001; MW test: *U* = 906.5, *P* = 0.002]; VOC [Welch’s *t*-test: *t*(52.7) = −3.92, *P* < 0.001; MW test: *U* = 938.5, *P* < 0.001]; MORPH [Welch’s *t*-test: *t*(55.8) = −2.43, *P* = 0.009; MW test: *U* = 831.5, *P* = 0.020]; SEN [Welch’s *t*-test: *t*(49.0) = −2.34, *P* = 0.012; MW test: *U* = 840.0, *P* = 0.015]; EFFIC [Welch’s *t*-test: *t*(53.9) = −3.41, *P* < 0.001; MW test: *U* = 890.0, *P* = 0.003]; RCOMP [Welch’s *t*-test: *t*(47.5) = −2.3, *P* = 0.013; MW test: *U* = 828.0, *P* = 0.022].

### Primary predictors

**Figure 3:**
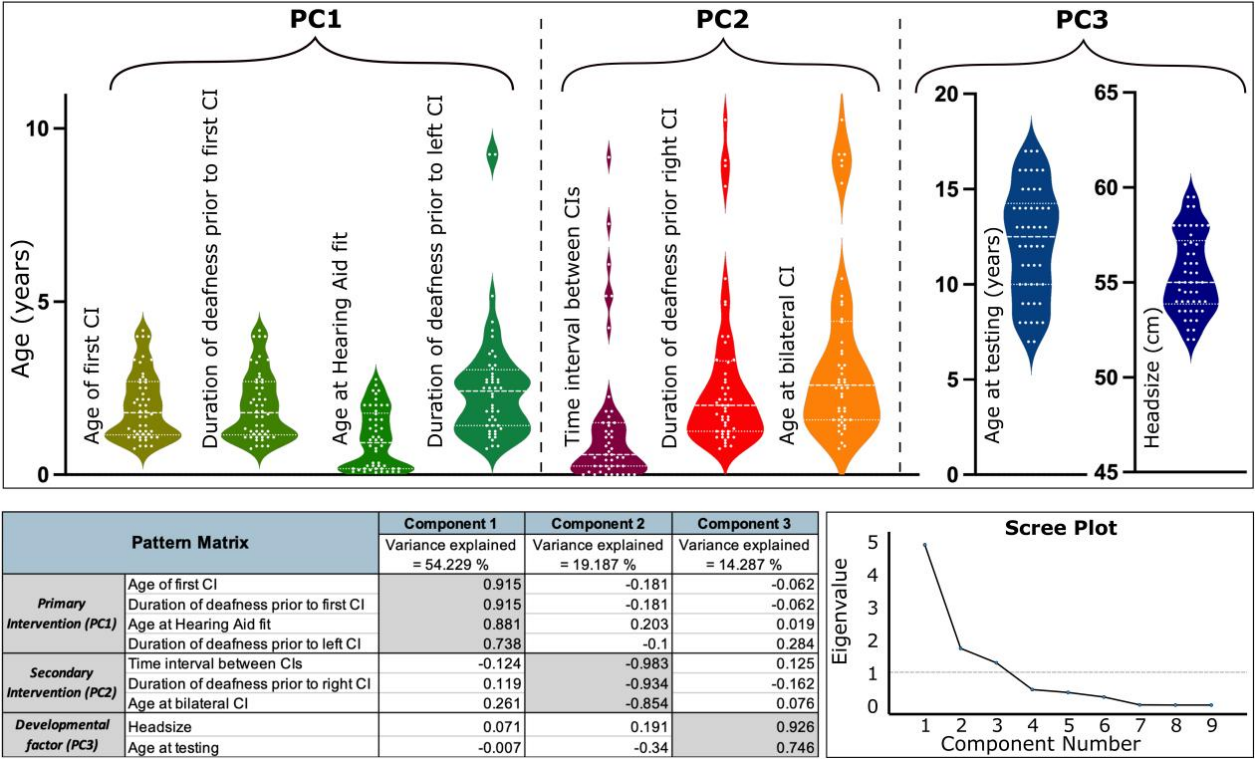
**Principal component analysis.** Output of the PCA analysis conducted with all clinical measures considered. The pattern matrix shows the component loadings for the three components selected. Scree plot justifying the selection of three components for the study (eigenvalues for 4th or higher components are <1). The total number of subjects used in the analysis was 50 (children with CI).

The PCA analysis resulted in three meaningful components (scree plot in Fig. 3) which explained 54.3%, 19.2% and 14.3% of the variance respectively (pattern matrix in Fig. 3). The first component (PC1) referred to hereafter as *primary intervention*, encompassed the age of first CI, duration of deafness prior to first CI, age of hearing aid fit and duration of deafness prior to left CI whereas the second component (PC2) referred to hereafter as *secondary intervention* included time between the bilateral CI, duration of deafness prior to right CI and age of bilateral CI and the third component (PC3) referred to hereafter as *development factor* included the head size and age at testing (violin plots in Fig. 3).

### Mediating effects of connectivity

The effects of PC1 and PC3 on the outcome variables for children with CI compared with their TH peers were influenced by a number of reading network connections, some of which were unique to each component and some that were common to both. There were no significant mediating effects of connectivity for PC2.

#### Primary intervention factor (PC1)

**Figure 4:**
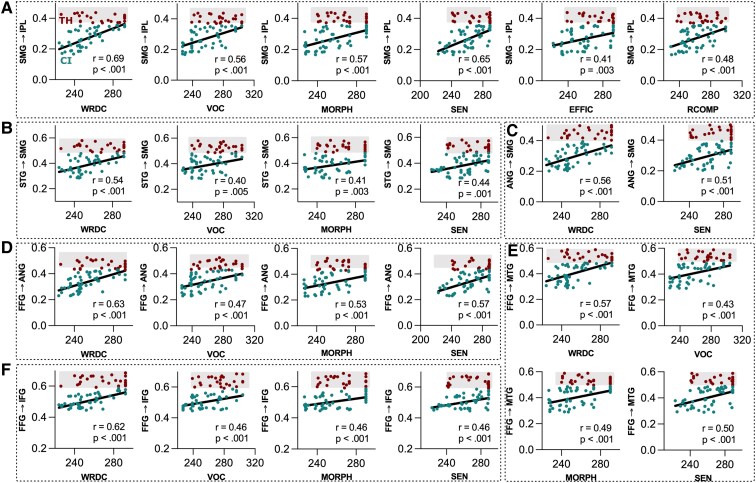
**Connectivity strength and reading outcome.** Scatterplots showing the relationship between the RISE scores to the TPDC-based connectivity values in the connections which showed significant mediation for the effect of age of intervention. The correlation (best-fit line) is shown here for the CI group; the TH datapoints (which do not show a relationship) are included for highlighting the connectivity strength differences (higher overall for TH than for the children with CI) between TH children and those with cochlear implants. (**A**) The relationship between the connectivity SMG to IPL to RISE subtests; (**B**) It for the connectivity STG to SMG; (**C**) ANG to SMG; (**D**) FFG to ANG; (**E**) FFG to MTG and (**F**) FFG to IFG. Pearson correlation was conducted to assess the correlation for the 50 subjects (children with CI).

The significant mediation effects (indirect effects) for the primary intervention factor (PC1) were associated with negative coefficients indicating that later hearing intervention was associated with greater reductions in connection strength (see Supplementary Tables 1–6 for detailed results). Out of 252 possible connections, there were 47 significant connections for the six reading subtests with the following distribution: WRDC—13, VOC—8, MORPH—10, SEN—14, EFFIC—1 and RCOMP—1. A graphical summary of the significant mediating connections for each subtest is provided in Supplementary Fig. 2. The influence of connectivity strength to the individual reading outcomes in children with CI for the significant connections of PC1 are shown in Fig. 4.

#### Developmental factor (PC3)

The significant mediation effects (indirect effects) for the development factor (PC3) were also negative indicating the impact of hearing loss on the development factor was associated also with an overall reduction in connection strength (see Supplementary Tables 7–12 for detailed results). Out of 252 possible connections, there were 102 significant connections for the six reading subtests with the following distribution: WRDC—16, VOC—17, MORPH—20, SEN—19, EFFIC—14 and RCOMP—16. A graphical summary of the significant mediating connections for each subtest is provided in Supplementary Fig. 3.

**Figure 5:**
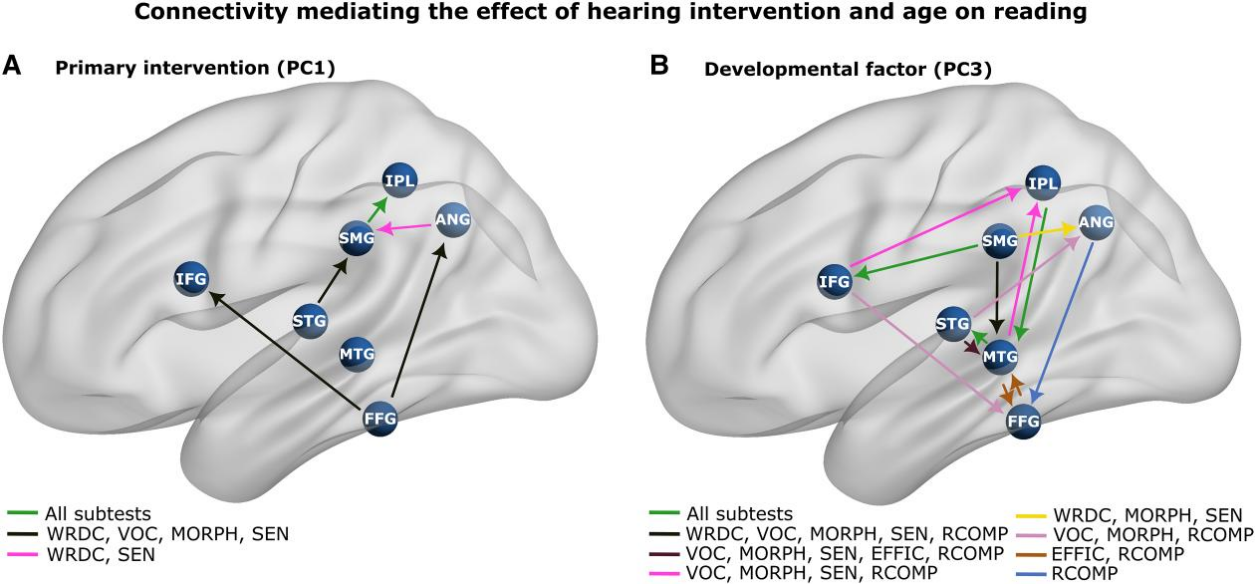
**Connectivity mediating the reading outcome.** Significant connection in the reading network mediating the effect of the primary intervention (PC1) and developmental factor (PC3) to different aspects of reading. (**A**) The significant connections which are unique to hearing intervention with differential effects on different aspects of reading (colour coded). (**B**) The connections which are unique to developmental factors with differential effects on different aspects of reading (colour coded). Here, the blue nodes indicate regions of reading network—IFG, STG, MTG, FFG, SMG, IPL, ANG selected for the study. Different aspects of reading are captured by the subtest of RISE as WRDC, VOC, MORPH, SEN, EFFIC and RCOMP. Structural equation modelling (SEM) was used for conducting the mediation analysis using the connectivity values from 50 subjects.

A summary comparison of the connectivity results for the different reading subtests for each factor (PC1 and PC3) is presented in Fig. 5. For PC1, the connectivity between SMG and IPL was associated with all subtests; the connections from the FFG to ANG, FFG to IFG and STG to SMG were associated with WRDC, VOC, MORPH and SEN; the ANG to SMG connection was associated with WRDC and SEN only (Fig. 5A). For PC3, there were more connections than for PC1, with connections from IPL to MTG, MTG to STG and SMG to IFG mediating all subtests. In addition, there were more reciprocal interactions compared with PC1 with connections to and from MTG to FFG, STG and IPL (Fig. 5B).

### First hearing intervention and hearing age

**Figure 6:**
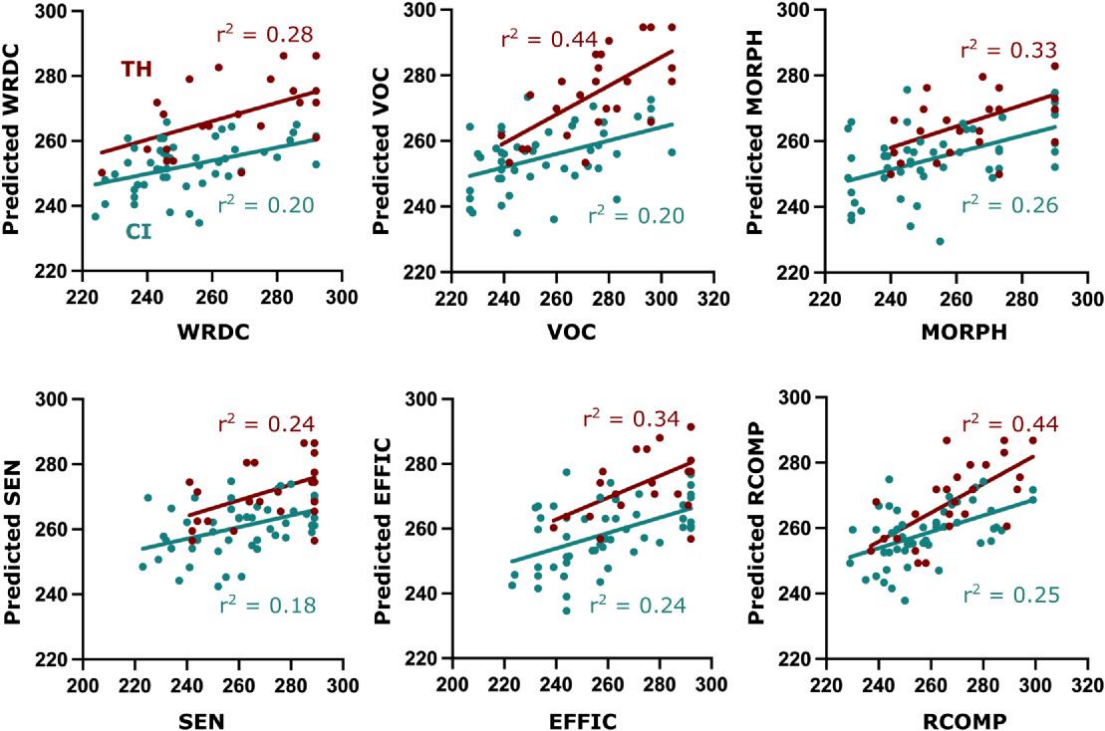
**Impact of hearing age on reading development.** Regression plots for simple linear regression used to test if age significantly predicted RISE subtests for TH children and multivariate regression to test if hearing age and age of hearing aid fit significantly predicted RISE subtests in children with CIs. The figure shows the comparison of the fitted regression model and the difference between the slopes of the predictors in children with CI (*N* = 50) and TH (*N* = 25).

Our final analysis focused on the variables within PC1 and PC3 to determine their influence on behavioural outcomes. We explored correlations within each component, identifying key variables for each reading measure (Supplementary Table 13). For PC1, the age at which the first hearing aid was fitted emerged as the most robust and significant predictor across multiple reading subtests notably WRDC, MORPH, SEN and RCOMP compared with the other primary intervention measures. In contrast, for PC3, hearing age, showed the strongest correlations (Supplementary Table 13), significantly affecting VOC, MORPH, EFFIC and RCOMP measures. Multiple regression analyses were conducted using these two key predictors to assess their impact on reading outcomes. Figure 6 illustrates the predicted regression lines for each subtest, comparing children with CI and TH peers. For children with CI, the regression result indicated that the predictors (i.e. hearing age and age of hearing aid fit) explained 20.3% of the variation in WRDC [*F*(2,47) = 5.97, *P* = 0.005], 20.4% of the variation in VOC [*F*(2,47) = 6.01, *P* = 0.005], 25.9% of the variation in MORPH [*F*(2,47) = 8.23, *P* < 0.001], 18% of the variation in SEN [*F*(2,47) = 5.15, *P* = 0.01], 23.7% of the variation in EFFIC [*F*(2,47) = 7.32, *P* = 0.002], 24.9% of the variation in RCOMP [*F*(2,47) = 7.81, *P* = 0.001]. For TH children, the predictor (age) explained 28.4% of the variation in WRDC [*F*(1,23) = 9.13, *P* = 0.006], 43.8% of the variation in VOC [*F*(1,23) = 17.92, *P* < 0.001], 32.5% of the variation in MORPH [*F*(1,23) = 11.06, *P* = 0.003], 24.2% of the variation in SEN [*F*(1,23) = 7.33, *P* = 0.013], 33.7% of the variation in EFFIC [*F*(1,23) = 11.69, *P* = 0.002], 44% of the variation in RCOMP [*F*(1,23) = 18.09, *P* < 0.001]. The regression analyses revealed lower slopes for all subtests in children with CIs when compared with TH peers (Fig. 6), highlighting the complex interplay of delayed development and intervention timing effects of deafness on reading abilities.

## Discussion

The current study examines factors associated with reading-related outcomes in children with CI focusing on the effects of intervention timing and age-dependent changes. We investigated neural connectivity within the canonical reading network by comparing it to TH children, using haemodynamic data obtained under naturalistic stimuli. Our analysis revealed reduced connectivity within the reading network for several foundational reading skills, which correlated with the timing of initial intervention. Furthermore, by assessing network-level connectivity based on participant age, we observed reduced connectivity associated with all examined reading skills. These findings underscore the significance of network-level connectivity in influencing the success of reading skill development in children with CI.

### Effects of intervention on reading development

Among children with CIs, we observed significant variability in reading scores, with some demonstrating age-appropriate skills while others lagged behind. The development of reading abilities is closely linked to neural connectivity within and across brain regions specialized for reading.^37,99^ Specifically, reading proficiency has been associated with the modulation of connections between the IFG and the inferior parietal lobe (IPL), as well as bidirectional modulation involving the IPL and the medial frontal gyrus,^100^ and between the FFG and language-related areas in the IPL and IFG.^101^ Further, connectivity involving the STG and the SMG has been linked to phonological processing and the mapping between orthography and phonology.^102,103^ Functionally, the connections among the STG, SMG and the ANG play crucial roles in phonological and word-processing tasks, such as linking phonemes into graphemes, and processing word meaning (cf. Kearns *et al.*^104^). The visual analysis of written words and access to its meaning^37,40,105,106^ including letter and word recognition, sight word recognition and semantic/meaning processing engages the FFG and the middle temporal gyrus (MTG) (cf. Dehaene and Cohen^107^). Additionally, parts of the IFG are implicated in storing and processing phonological structure.^108^

Our findings suggest that network-level connectivity differences in children with CI, when compared with those with TH, affect foundational reading abilities. Early hearing intervention on connectivity appears to have a pronounced impact on word decoding, VOC, MORPH and SEN (linked result: Fig. 5 and supplementary Fig. 1, mediation results of PC1). Specifically, we found reductions in connectivity for pathways critical for WRDC, such as those from the ANG to SMG and a sequential pathway from the STG to SMG to IPL. Furthermore, VOC and MORPH, which are features intrinsically linked to memory and comprehension of linguistic constructs, showed a reduction in connectivity strength originating from the FFG. This connectivity, principally affecting areas like the IFG, MTG and ANG highlights the strong influence of auditory processing and deprivation on the functioning of the FFG.

The most consistent finding for the timing of primary intervention was reduced connectivity between the SMG and IPL, modulating the efficacy of all reading subtests. These two regions are integral in contribution to phonological and semantic aspects of word processing^108-110^ including integrating orthography and phonology,^111,112^ visual word recognition^113^ and reading comprehension. Moreover, the posterior portion of the IPL and its connections with the AG, is a heteromodal convergence area involved in a number of cognitive operations including semantic memory.^114^ Interestingly, connectivity among the seven ROIs studied was less implicated in reading efficiency and comprehension, suggesting the potential involvement of additional brain areas beyond the primary reading network is important for the children with CI and is possibly impacted by intervention timing as well. This is a finding that resonates with other observations not necessarily tied to reading but to language and learning.^115-119^

### Effects in dynamic functional connectivity and age

A secondary focus of this study was on the overall impact of deafness on the development of neural connectivity within the reading network in children with CI. Here, we observed a substantial negative impact on network-level connectivity that was strongly associated with the fundamental reading abilities. Consistent with the general observation that children born deaf experience developmental delays, we also found significant differences in reading network connectivity attributable to developmental factors (linked result: Fig. 5 and supplementary Fig. 2, mediation results of PC3). Overall, there were more distinct connectivity differences related to development than for initial hearing intervention (a greater number of connections significantly mediating the effect of PC3 than PC1). Reductions in connectivity strength were observed for most connections and all assessed reading skills, with notable differences when compared with the effects of intervention timing. For the four subtests assessing basic reading fundamentals—WRDC, VOC, MORPH and SEN—many network nodes exhibited reductions in connectivity due to developmental factors, contrasting with those affected by intervention timing. For instance, SMG-IPL connectivity, was impacted across all subskills by intervention timing but was unaffected by developmental factor. Conversely, reduced connection strength to and from the IFG played a prominent role for the developmental factor but not for the initial intervention. It is noteworthy that MTG, a crucial node linking the dorsal and ventral streams for reading, was heavily impacted by development but not by initial hearing intervention. Given the role of MTG as an important region for linking phonological and semantic functions, the reduction in connection strength may be an important biomarker for overall reading difficulty. Interestingly, the two measures of reading comprehension (EFFIC and RCOMP) were more adversely affected by development than by the timing of the initial intervention. This, alongside the multiple regression analyses revealing slope and intercept differences across all reading subtests (see Fig. 5), suggests that some children with CI face persistent reading challenges that may not remediate with time.

To highlight a few more points which further strengthen the robustness and nonrandom nature of the findings. First, we conducted the same analysis (252 separate analyses) for TH children (using age instead of PC1) and none of the direct or indirect effects survived the significance threshold suggesting that the significant mediations observed in the CI group are likely driven by the unique characteristics of their connectivity profiles. As shown in Fig. 4, there is significant intersubject variability in connectivity strength across both groups. However, the children with TH consistently exhibit significantly higher connectivity strengths than the children with CI. The results suggest that the mediation effects in the CI group are influenced by differences in connectivity strength rather than the presence or absence of specific connections.

### The neural effects of hearing deprivation and prosthetic intervention

Neural connections within and between brain areas begin to form in utero and evolve through multiple developmental stages. Early sensory deprivation limits both the development of these connections both prenatally and postnatally from environmental interactions (cf. Kral *et al.*^4^). Functional neuroimaging studies have identified several neural changes in children born with profound bilateral sensorineural hearing loss, including reduced functional connectivity and reorganization of functional networks.^120-123^ For children born with profound bilateral sensorineural hearing loss prior to implantation, reduced functional connectivity is one consistent finding along with a reorganization of functional networks. After prosthetic intervention, children with hearing loss undergo neural changes that can restore some adaptive changes that occurred prior to intervention. A significant factor in determining the form and extent of the adaptation is the timing of the intervention relative to critical periods of development.^124^ Based on our observation that almost all children with CI displayed reduced connectivity (see Fig. 5) and deviated developmental trajectories (see Fig. 6), early sensory deprivation and/or neural adaptations appear to contribute to the reading abilities of children with CI. For those children whose reading abilities were within the age-appropriate range, the reduced connectivity suggests alternative pathways for achieving age-appropriate reading ability. These pathways, formed outside the traditional reading network may partially circumvent the limitations imposed by early sensory deprivation, suggesting the use of compensatory strategies (e.g. visual processing strategies) in deaf children with CI to support language and reading development.^125^ Our previous study using EEG under the same condition (watching the Inscapes video) did show that children with CI and different language abilities exhibit different cortical and subcortical activation patterns and connectivity, suggesting compensatory adaptations that either facilitate or impede language development.^12^ It appears that both good and struggling readers may rely to some extent on supplementary brain regions to compensate for the effects of early auditory deprivation. While this compensation may provide some benefit to the development of reading skills, the current data suggest that even among children with CI who demonstrate good reading abilities, these adaptations may not be sufficient to fully offset the impact of early hearing deprivation as children with CI as they transition from learning to read to reading to learn. As a result, it is important to emphasize the need for long term and comprehensive approaches to support the neural and cognitive development of children with CI, acknowledging the potential for and limitations of neural plasticity in the context of sensory deprivation.

## Conclusion

Congenital hearing deprivation results in changes to the developing brain connectome.^2-4,13,120,122,123,126-128^ The current findings provide evidence of the effects of such deprivation on connectome in brain regions associated with reading. Specifically, brain regions within the reading network exhibited reduced connectivity, with the strength of connectivity dependent on factors related to the timing of prosthetic intervention. Within our sample, earlier fitting of hearing aids and earlier age of first cochlear implantation were associated with increased connectivity strength. Additionally, the stronger connectivity in a number of reading-related brain regions was correlated with better reading ability.

Furthermore, as development progressed (age and increased time using the implanted device), notable improvements were seen in connectivity strength and reading scores. However, despite these gains, overall connectivity strength and reading scores only approximated those of the children with TH. Among the primary intervention factors (age of first CI and hearing aid, duration of deafness prior to first CI and duration of deafness prior to left CI), the most significant connection mediating all the reading subfunctions was between the SMG and the inferior parietal lobe—a subnetwork crucial for both semantic and phonological aspects for reading development.

The connectivity and interaction between brain regions play a crucial role in the identification of reading ability in children with CI. Despite its critical importance, research associated with changes in brain connections and their associated dynamics remains underexplored. The current study is one of the first to examine changes in the connectome in relation to reading outcomes in children with CIs. While the current study was exploratory given the limited data in the literature for children with CI, we were focused on the mechanism that mediated the interventional and developmental impact on language and reading skills. Specifically, we tested an assumption that differences were related to the strength of the connections within functional networks known to contribute to language and reading. Our previous work associated reading and language development with distinct neural dynamics in children with CI from those in children with TH.^126,129-131^ Differences in the connectome and the interactions among functional brain areas for language, reading and cognitive processing offer potential insights into the adaptive strategies that yield successful and less successful outcomes in children with CI. The method applied in the study could enable us to look into these connectomic differences and how they might be contributing to a range of outcomes. These findings are a critical complement to behavioural measures, providing potential targets for new habilitation approaches such as neurofeedback.

## Supplementary Material

fcaf086_Supplementary_Data

## Data Availability

Identifiable patients’ data used for the study cannot be shared because of the agreement signed with the participants. However, partially analysed, deidentified electrophysiological and behavioural data could be shared with appropriate request and IRB approval letter to the corresponding author. The relevant codes used in the study can be obtained in this link: https://github.com/nabinkrl/The-Neural-Characteristics-Influencing-Literacy-Outcome-in-Children-with-Cochlear-Implants.
