# Supplementary material for: The neural characteristics influencing literacy outcome in children with cochlear implants
Source: Brain Commun. 2025 Feb 21;7(2):fcaf086. doi: 10.1093/braincomms/fcaf086 (PMC11881800; doi:10.1093/braincomms/fcaf086)
Supplement: fcaf086_Supplementary_Data [file fcaf086_supplementary_data.pdf]

The Neural Characteristics Influencing Literacy Outcome in Children with Cochlear Implants

Nabin Koirala[1,2,3*], Jacy Manning[4], Sara Neumann[4], Chelsea Anderson[4], Mickael Deroche[5], Jace Wolfe[6], Kenneth Pugh[1,7], Nicole Landi[1,7], Muthuraman Muthuraman[8§], Vincent Gracco[1,9§]

[1] Child Study Center, School of Medicine, Yale University, New Haven, CT, USA
[2] Brain Imaging Research Core, University of Connecticut, Storrs, CT, USA
[3] Nathan Kline Institute for Psychiatric Research, Orangeburg, NY, USA
[4] Hearts for Hearing Foundation, Oklahoma City, OK, USA
[5] Department of Psychology, Concordia University, Montreal, QC, Canada
[6] Oberkotter Foundation, Philadelphia, PA, USA
[7] Department of Psychological Sciences, University of Connecticut, Storrs, CT, USA
[8] Department of Neurology, Universitätsklinikum Würzburg, Würzburg, Germany
[9] School of Communication Sciences and Disorders, McGill University, Montreal, QC, Canada

[§] Shared contribution

[*] Corresponding author
Nabin Koirala, PhD
Yale Child Study Center
Yale School of Medicine
300 George Street, New Haven, Connecticut 06511, USA
Email: nabin.koirala@yale.edu
Phone: +1 203 785 2527

Supplementary Material

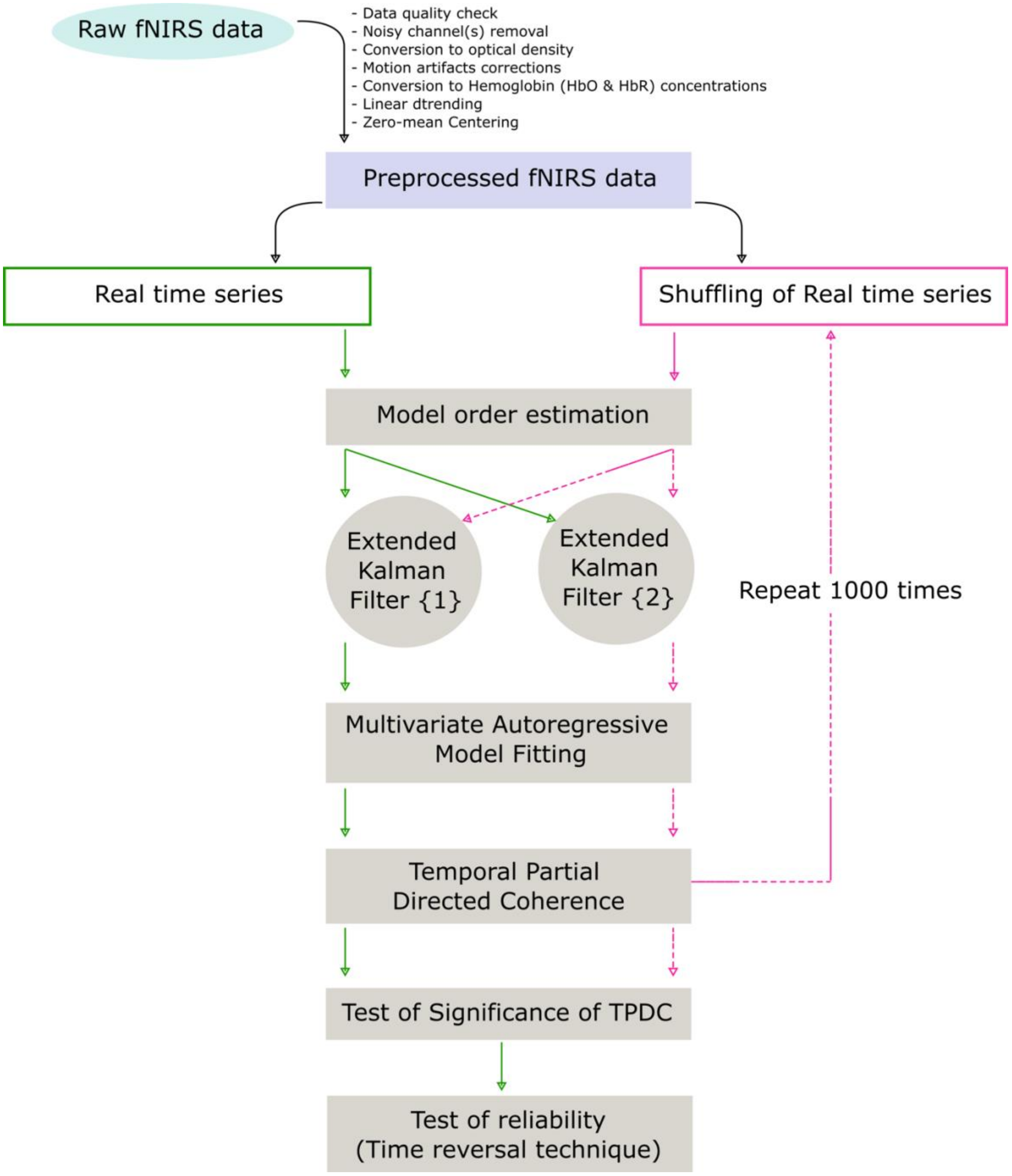

Supplementary Figure 1: Methods Flowchart. Flowchart detailing the steps used for computing directed functional connectivity using temporal partial directed coherence.

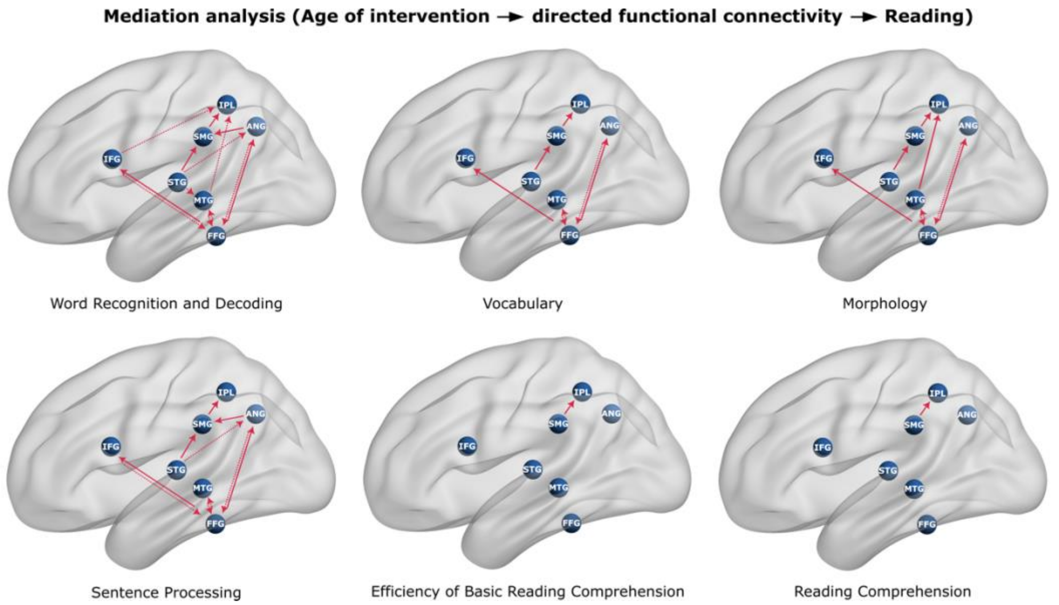

Supplementary Figure 2: Mediation results-PC1. Significant connection in the reading network mediating the effect of primary intervention (PC1) to different aspects of reading. The dotted lines indicate the connections which overlap with similar effect of developmental factor (PC3). The blue nodes indicate regions of reading network - Inferior frontal gyrus (IFG), Superior temporal gyrus (STG), Medial temporal gyrus (MTG), Fusiform gyrus (FFG), Supramarginal gyrus (SMG), Inferior parietal lobule (IPL), Angular Gyrus (AG) selected for the study. The red arrows indicate the direction of the functional connectivity. Note: The mediation analysis was conducted using structural equation modeling, see methods for more details. The total number of subjects used in the analysis was 50 (children with CI).

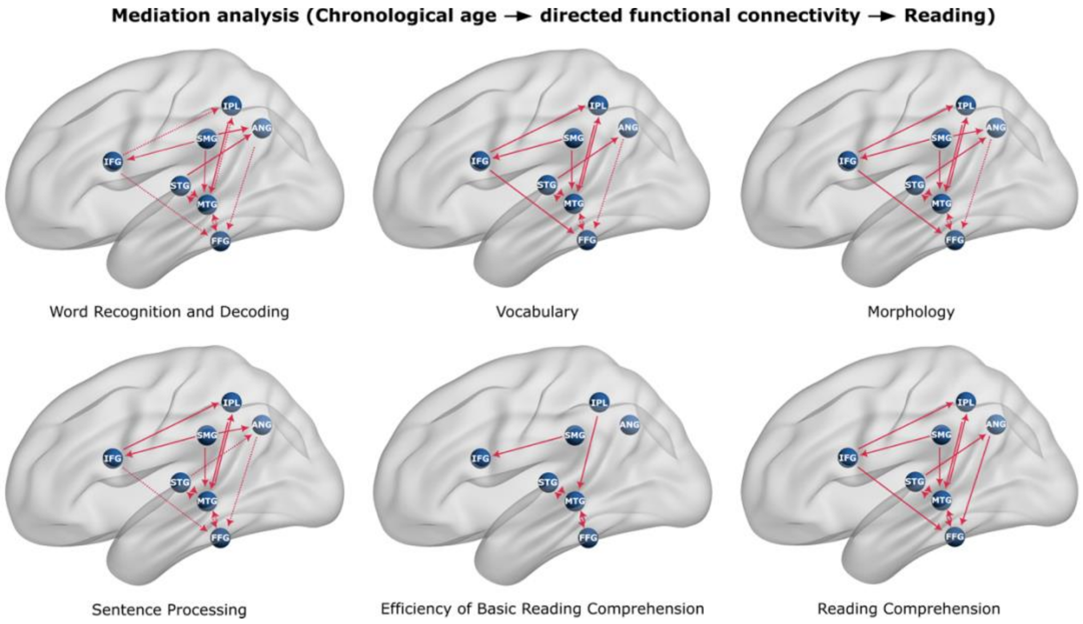

Supplementary Figure 3: Mediation results-PC3. Significant connection in the reading network mediating the effect of developmental factor (PC3) to different aspects of reading. The dotted lines indicate the connections which overlap with similar effect of age of intervention (PC1). Here, blue nodes indicate regions of reading network - Inferior frontal gyrus (IFG), Superior temporal gyrus (STG), Medial temporal gyrus (MTG), Fusiform gyrus (FFG), Supramarginal gyrus (SMG), Inferior parietal lobule (IPL), Angular Gyrus (AG) selected for the study. The red arrows indicate the direction of the functional connectivity. The mediation analysis was conducted using structural equation modeling, see methods for more details. The total number of subjects used in the analysis was 50 (children with CI).

Mediation analysis with PC1 and each subtest of RISE for children with CI

Connectivity	**Direct effect: TPDC ~ PC1 (a1)** *Estimate [SE]*	**Direct effect: WRDC ~ TPDC (b1)** *Estimate [SE]*	**Direct effect: WRDC ~ PC1 (c1)** *Estimate [SE]*	**Indirect effect: WRDC ~ PC1 + TPDC (c1′)** *Estimate [SE]*
SMG -> IPL	-0.02 [0.01]	10.49 [1.83]	-0.08 [0.11]	-0.26 [0.10]*
SMG -> ANG	-0.01 [0.00]	9.61 [4.55]	-0.26 [0.14]	-0.08 [0.06]
SMG -> STG	-0.01 [0.00]	-8.05 [5.56]	-0.39 [0.14]	0.06 [0.05]
SMG -> MTG	-0.02 [0.01]	5.84 [2.67]	-0.24 [0.14]	-0.10 [0.06]
SMG -> FFG	-0.00 [0.00]	2.66 [4.69]	-0.33 [0.14]	-0.01 [0.02]
SMG -> IFG	-0.02 [0.01]	8.35 [4.69]	-0.33 [0.14]	-0.01 [0.02]
IPL -> SMG	-0.00 [0.00]	-2.93 [4.54]	-0.33 [0.14]	-0.01 [0.02]
IPL -> ANG	-0.00 [0.01]	2.59 [4.39]	-0.33 [0.14]	-0.00 [0.01]
IPL -> STG	0.00 [0.00]	-4.11 [5.96]	-0.34 [0.14]	-0.00 [0.01]
IPL -> MTG	-0.02 [0.01]	7.23 [2.99]	-0.23 [0.14]	-0.11 [0.06]
IPL -> FFG	-0.01 [0.00]	-5.31 [4.44]	-0.38 [0.14]	-0.04 [0.04]
IPL -> IFG	0.00 [0.00]	-11.76 [4.43]	-0.30 [0.13]	-0.04 [0.05]
ANG -> SMG	-0.02 [0.01]	8.41 [2.10]	-0.17 [0.12]	-0.16 [0.10]*
ANG -> IPL	-0.00 [0.00]	-4.85 [4.67]	-0.35 [0.14]	0.02 [0.03]
ANG -> STG	-0.02 [0.01]	6.57 [2.68]	-0.23 [0.14]	-0.11 [0.01]
ANG -> MTG	0.00 [0.01]	3.03 [4.36]	-0.34 [0.14]	0.00 [0.01]
ANG -> FFG	-0.03 [0.01]	9.92 [2.07]	-0.07 [0.12]	-0.26 [0.10]*
ANG -> IFG	-0.00 [0.00]	-0.72 [4.61]	-0.34 [0.14]	-0.00 [0.01]
STG -> SMG	-0.03 [0.01]	7.93 [2.23]	-0.13 [0.13]	-0.21 [0.09]*
STG -> IPL	-0.00 [0.00]	5.14 [4.85]	-0.33 [0.14]	-0.01 [0.02]
STG -> ANG	-0.03 [0.01]	6.37 [2.49]	-0.18 [0.14]	-0.16 [0.08]*
STG -> MTG	-0.02 [0.01]	8.51 [1.88]	-0.17 [0.12]	-0.17 [0.08]*
STG -> FFG	0.01 [0.00]	-2.53 [4.51]	-0.33 [0.14]	-0.01 [0.02]
STG -> IFG	0.01 [0.00]	-3.75 [5.11]	-0.37 [0.14]	-0.03 [0.04]
MTG -> SMG	0.01 [0.01]	-0.78 [4.41]	-0.33 [0.14]	-0.01 [0.03]
MTG -> IPL	-0.02 [0.01]	8.55 [1.96]	-0.16 [0.12]	-0.17 [0.08]*
MTG -> ANG	-0.00 [0.00]	-2.95 [4.70]	-0.35 [0.14]	-0.01 [0.02]
MTG -> STG	-0.03 [0.01]	5.41 [2.32]	-0.19 [0.14]	-0.14 [0.08]
MTG -> FFG	-0.03 [0.01]	8.42 [2.10]	-0.13 [0.13]	-0.21 [0.09]*
MTG -> IFG	0.00 [0.00]	2.62 [4.66]	-0.35 [0.14]	-0.01 [0.02]
FFG -> SMG	-0.00 [0.00]	4.54 [4.73]	-0.33 [0.14]	-0.01 [0.02]
FFG -> IPL	-0.00 [0.00]	5.81 [4.91]	-0.34 [0.14]	-0.00 [0.02]

FFG -> ANG	-0.03 [0.01]	8.81 [2.04]	-0.12 [0.13]	-0.21 [0.01]*
FFG -> STG	-0.00 [0.00]	-1.96 [4.47]	-0.34 [0.14]	0.00 [0.01]
FFG -> MTG	-0.03 [0.01]	7.99 [2.15]	-0.08 [0.14]	-0.25 [0.09]*
FFG -> IFG	-0.02 [0.01]	14.59 [3.10]	-0.09 [0.12]	-0.25 [0.09]*
IFG -> SMG	-0.00 [0.00]	-5.16 [4.83]	-0.36 [0.14]	-0.02 [0.03]
IFG -> IPL	-0.03 [0.01]	6.51 [2.13]	-0.16 [0.14]	-0.17 [0.08]*
IFG -> ANG	-0.00 [0.00]	-4.67 [5.40]	-0.35 [0.14]	-0.01 [0.02]
IFG -> STG	-0.02 [0.01]	8.52 [2.31]	-0.20 [0.13]	-0.14 [0.08]
IFG -> MTG	-0.00 [0.00]	-3.66 [4.74]	-0.34 [0.14]	-0.01 [0.02]
IFG -> FFG	-0.02 [0.01]	7.00 [2.25]	-0.18 [0.13]	-0.16 [0.08]*

Supplementary table 1: The table shows all effects (direct and indirect) of mediation analysis for age of intervention (PC1) as predictors, temporal directed partial coherence as a measure for directed functional connectivity as mediators and word recognition and decoding (WRDC) subtest of Reading Inventory and Scholastic Evaluation (RISE) as the output measures. The values presented are regression estimates (unstandardized beta values) and standard error. All significant ($p < 0.05$, corrected using BH procedure) indirect effects are highlighted with the red font and asterisks and the corresponding significant direct effects are highlighted with red font.

**RISE – Vocabulary (VOC)**				
**Connectivity**	**Direct effect: TPDC ~ PC1 (a1)** *Estimate [SE]*	**Direct effect: VOC ~ TPDC (b1)** *Estimate [SE]*	**Direct effect: VOC ~ PC1 (c1)** *Estimate [SE]*	**Indirect effect: VOC ~ PC1 + TPDC (a1*b1)** *Estimate [SE]*
SMG -> IPL	-0.02 [0.01]	9.38 [2.10]	0.06 [0.13]	-0.23 [0.01]*
SMG -> ANG	-0.01 [0.00]	2.50 [4.99]	-0.15 [0.15]	-0.02 [0.04]
SMG -> STG	-0.01 [0.00]	-8.31 [5.88]	-0.23 [0.15]	0.06 [0.05]
SMG -> MTG	-0.02 [0.01]	3.55 [2.89]	-0.11 [0.15]	-0.06 [0.06]
SMG -> FFG	-0.00 [0.00]	3.34 [4.92]	-0.16 [0.14]	-0.01 [0.02]
SMG -> IFG	-0.02 [0.01]	7.19 [2.23]	-0.04 [0.14]	-0.13 [0.07]
IPL -> SMG	-0.00 [0.00]	-1.80 [4.77]	-0.17 [0.14]	-0.00 [0.01]
IPL -> ANG	-0.00 [0.01]	-2.82 [4.61]	-0.18 [0.14]	-0.00 [0.01]
IPL -> STG	0.00 [0.00]	0.29 [6.29]	-0.17 [0.14]	0.00 [0.01]
IPL -> MTG	-0.02 [0.01]	4.39 [3.27]	-0.11 [0.15]	-0.07 [0.06]
IPL -> FFG	-0.01 [0.00]	-4.51 [4.69]	-0.20 [0.15]	-0.03 [0.04]
IPL -> IFG	0.00 [0.00]	-11.65 [4.69]	-0.13 [0.14]	-0.04 [0.05]
ANG -> SMG	-0.02 [0.01]	6.90 [2.33]	-0.04 [0.14]	-0.13 [0.10]
ANG -> IPL	-0.00 [0.00]	1.08 [4.95]	-0.17 [0.14]	-0.00 [0.02]
ANG -> STG	-0.02 [0.01]	6.27 [2.84]	-0.07 [0.14]	-0.10 [0.06]
ANG -> MTG	0.00 [0.01]	1.83 [4.60]	-0.17 [0.14]	0.00 [0.01]
ANG -> FFG	-0.03 [0.01]	7.93 [2.38]	0.04 [0.14]	-0.21 [0.09]*
ANG -> IFG	-0.00 [0.00]	0.64 [4.84]	-0.17 [0.14]	-0.00 [0.01]
STG -> SMG	-0.03 [0.01]	6.67 [2.46]	0.00 [0.15]	-0.17 [0.08]*
STG -> IPL	-0.00 [0.00]	3.47 [5.13]	-0.17 [0.14]	-0.01 [0.02]
STG -> ANG	-0.03 [0.01]	3.53 [2.79]	-0.09 [0.16]	-0.09 [0.07]
STG -> MTG	-0.02 [0.01]	5.96 [2.21]	-0.06 [0.14]	-0.12 [0.07]
STG -> FFG	0.01 [0.00]	0.94 [4.75]	-0.18 [0.15]	0.00 [0.02]
STG -> IFG	-0.01 [0.00]	-7.45 [5.29]	-0.23 [0.15]	0.06 [0.05]
MTG -> SMG	0.01 [0.01]	1.82 [4.63]	-0.18 [0.15]	0.01 [0.03]

MTG -> IPL	-0.02 [0.01]	7.51 [2.18]	-0.02 [0.13]	-0.15 [0.08]
MTG -> ANG	-0.00 [0.00]	0.13 [4.96]	-0.17 [0.14]	-0.00 [0.01]
MTG -> STG	-0.03 [0.01]	3.42 [2.52]	-0.08 [0.16]	-0.09 [0.07]
MTG -> FFG	-0.03 [0.01]	6.38 [2.39]	-0.02 [0.15]	-0.16 [0.08]*
MTG -> IFG	0.00 [0.00]	0.34 [4.91]	-0.17 [0.14]	0.00 [0.02]
FFG -> SMG	-0.00 [0.00]	5.55 [4.95]	-0.16 [0.14]	-0.02 [0.03]
FFG -> IPL	-0.00 [0.00]	4.50 [5.19]	-0.17 [0.14]	-0.00 [0.02]
FFG -> ANG	-0.02 [0.01]	7.24 [2.31]	0.00 [0.14]	-0.18 [0.08]*
FFG -> STG	-0.00 [0.00]	-2.05 [4.69]	-0.17 [0.14]	0.00 [0.01]
FFG -> MTG	-0.03 [0.01]	6.36 [2.39]	0.03 [0.15]	-0.20 [0.09]*
FFG -> IFG	-0.02 [0.01]	12.06 [3.54]	0.03 [0.14]	-0.20 [0.09]*
IFG -> SMG	-0.00 [0.00]	-6.16 [5.06]	-0.19 [0.14]	0.03 [0.03]
IFG -> IPL	-0.03 [0.01]	4.01 [2.38]	-0.06 [0.15]	-0.11 [0.07]
IFG -> ANG	-0.00 [0.00]	0.96 [5.70]	-0.17 [0.14]	-0.00 [0.01]
IFG -> STG	-0.02 [0.01]	6.86 [2.57]	-0.06 [0.14]	-0.11 [0.07]
IFG -> MTG	-0.00 [0.00]	-4.51 [4.96]	-0.18 [0.14]	0.00 [0.02]
IFG -> FFG	-0.02 [0.01]	5.91 [2.45]	-0.03 [0.15]	-0.13 [0.07]

Supplementary table 2: The table shows all effects (direct and indirect) of mediation analysis for age of intervention (PC1) as predictors, temporal directed partial coherence as a measure for directed functional connectivity as mediators and word recognition and decoding (VOC) subtest of Reading Inventory and Scholastic Evaluation (RISE) as the output measures. The values presented are regression estimates (unstandardized beta values) and standard error. All significant ($p < 0.05$, corrected using BH procedure) indirect effects are highlighted with the red font and asterisks and the corresponding significant direct effects are highlighted with red font.

**RISE – Morphology (MORPH)**				
**Connectivity**	**Direct effect: TPDC ~ PC1 (a1)** *Estimate [SE]*	**Direct effect: MORPH ~ TPDC (b1)** *Estimate [SE]*	**Direct effect: MORPH ~ PC1 (c1)** *Estimate [SE]*	**Indirect effect: MORPH ~ PC1 + TPDC (a1*b1)** *Estimate [SE]*
SMG -> IPL	-0.02 [0.01]	9.46 [2.09]	0.03 [0.13]	-0.23 [0.09]*
SMG -> ANG	-0.01 [0.00]	4.18 [4.97]	-0.16 [0.15]	-0.04 [0.05]
SMG -> STG	-0.01 [0.00]	-6.42 [5.91]	-0.25 [0.15]	0.05 [0.05]
SMG -> MTG	-0.02 [0.01]	5.34 [2.83]	-0.11 [0.15]	-0.09 [0.06]
SMG -> FFG	-0.00 [0.00]	0.46 [4.95]	-0.19 [0.14]	-0.00 [0.01]
SMG -> IFG	-0.02 [0.01]	7.97 [2.18]	-0.06 [0.13]	-0.14 [0.08]
IPL -> SMG	0.00 [0.00]	-2.31 [4.77]	-0.19 [0.14]	-0.01 [0.02]
IPL -> ANG	-0.00 [0.01]	2.92 [4.61]	-0.20 [0.14]	0.00 [0.01]
IPL -> STG	0.00 [0.00]	-6.82 [6.22]	-0.19 [0.14]	-0.00 [0.02]
IPL -> MTG	-0.02 [0.01]	6.85 [3.19]	-0.09 [0.15]	-0.10 [0.06]
IPL -> FFG	-0.01 [0.00]	-5.11 [4.69]	-0.23 [0.15]	-0.04 [0.04]
IPL -> IFG	0.00 [0.00]	-12.01 [4.68]	-0.16 [0.14]	-0.04 [0.05]
ANG -> SMG	-0.02 [0.01]	7.88 [2.26]	-0.05 [0.14]	-0.15 [0.08]
ANG -> IPL	-0.00 [0.00]	-4.39 [4.91]	-0.21 [0.14]	0.01 [0.02]
ANG -> STG	-0.02 [0.01]	7.06 [2.80]	-0.08 [0.14]	-0.11 [0.07]
ANG -> MTG	0.00 [0.01]	2.03 [4.60]	-0.19 [0.14]	0.00 [0.01]
ANG -> FFG	-0.03 [0.01]	8.96 [2.31]	0.04 [0.14]	-0.24 [0.09]*
ANG -> IFG	0.00 [0.00]	-0.23 [4.84]	-0.19 [0.14]	-0.00 [0.00]
STG -> SMG	-0.03 [0.01]	6.78 [2.46]	-0.02 [0.15]	-0.18 [0.08]*

STG -> IPL	-0.00 [0.00]	3.51 [5.13]	-0.19 [0.14]	-0.01 [0.02]
STG -> ANG	-0.03 [0.01]	5.15 [2.69]	-0.07 [0.15]	-0.13 [0.08]
STG -> MTG	-0.02 [0.01]	7.09 [2.14]	-0.06 [0.13]	-0.14 [0.08]
STG -> FFG	0.01 [0.00]	0.29 [4.76]	-0.20 [0.15]	-0.00 [0.02]
STG -> IFG	0.01 [0.00]	-5.03 [5.36]	-0.24 [0.15]	0.04 [0.05]
MTG -> SMG	0.01 [0.01]	1.30 [4.64]	-0.21 [0.15]	0.01 [0.03]
MTG -> IPL	-0.02 [0.01]	8.24 [2.13]	-0.03 [0.13]	-0.17 [0.08]*
MTG -> ANG	-0.00 [0.00]	2.13 [4.96]	-0.19 [0.14]	-0.01 [0.02]
MTG -> STG	-0.03 [0.01]	5.24 [2.46]	-0.06 [0.15]	-0.14 [0.08]
MTG -> FFG	-0.03 [0.01]	7.77 [2.29]	-0.01 [0.14]	-0.19 [0.09]*
MTG -> IFG	0.00 [0.00]	2.22 [4.90]	-0.21 [0.14]	-0.01 [0.02]
FFG -> SMG	-0.00 [0.00]	0.83 [5.01]	-0.19 [0.14]	-0.00 [0.02]
FFG -> IPL	-0.00 [0.00]	1.01 [5.24]	-0.20 [0.14]	-0.00 [0.00]
FFG -> ANG	-0.02 [0.01]	8.19 [2.24]	-0.00 [0.14]	-0.20 [0.08]*
FFG -> STG	-0.00 [0.00]	-0.30 [4.71]	-0.20 [0.14]	0.00 [0.01]
FFG -> MTG	-0.03 [0.01]	7.58 [2.32]	0.04 [0.14]	-0.24 [0.09]*
FFG -> IFG	-0.02 [0.01]	11.59 [3.58]	-0.00 [0.14]	-0.20 [0.09]*
IFG -> SMG	-0.00 [0.00]	-3.42 [5.12]	-0.21 [0.14]	-0.01 [0.03]
IFG -> IPL	-0.03 [0.01]	5.27 [2.33]	-0.06 [0.15]	-0.14 [0.08]
IFG -> ANG	-0.00 [0.00]	-4.69 [5.66]	-0.21 [0.14]	0.01 [0.02]
IFG -> STG	-0.02 [0.01]	6.79 [2.57]	-0.09 [0.14]	-0.11 [0.06]
IFG -> MTG	-0.00 [0.00]	-8.61 [4.85]	-0.21 [0.14]	-0.01 [0.04]
IFG -> FFG	-0.02 [0.01]	6.48 [2.43]	-0.05 [0.14]	-0.15 [0.08]

Supplementary table 3: The table shows all effects (direct and indirect) of mediation analysis for age of intervention (PC1) as predictors, temporal directed partial coherence as a measure for directed functional connectivity as mediators and morphology (MORPH) subtest of Reading Inventory and Scholastic Evaluation (RISE) as the output measures. The values presented are regression estimates (unstandardized beta values) and standard error. All significant ($p < 0.05$, corrected using BH procedure) indirect effects are highlighted with the red font and asterisks and the corresponding significant direct effects are highlighted with red font.

**RISE – Sentence Processing (SEN)**				
**Connectivity**	**Direct effect: TPDC ~ PC1 (a1)** *Estimate [SE]*	**Direct effect: SEN ~ TPDC (b1)** *Estimate [SE]*	**Direct effect: SEN ~ PC1 (c1)** *Estimate [SE]*	**Indirect effect: SEN ~ PC1 + TPDC (a1*b1)** *Estimate [SE]*
SMG -> IPL	-0.02 [0.01]	10.53 [1.95]	-0.01 [0.12]	-0.26 [0.10]*
SMG -> ANG	-0.01 [0.00]	12.63 [4.59]	-0.16 [0.14]	-0.11 [0.07]
SMG -> STG	-0.01 [0.00]	-2.72 [5.90]	-0.29 [0.14]	0.02 [0.05]
SMG -> MTG	-0.02 [0.01]	6.21 [2.76]	-0.16 [0.14]	-0.11 [0.06]
SMG -> FFG	-0.00 [0.00]	1.95 [4.89]	-0.26 [0.14]	-0.01 [0.02]
SMG -> IFG	-0.02 [0.01]	9.21 [2.04]	-0.10 [0.12]	-0.17 [0.09]
IPL -> SMG	-0.00 [0.00]	0.45 [4.74]	-0.27 [0.14]	0.00 [0.01]
IPL -> ANG	-0.00 [0.01]	1.10 [4.58]	-0.27 [0.14]	-0.00 [0.01]
IPL -> STG	0.00 [0.00]	-4.54 [6.19]	-0.27 [0.14]	-0.00 [0.02]
IPL -> MTG	-0.02 [0.01]	6.96 [3.14]	-0.16 [0.14]	-0.11 [0.06]
IPL -> FFG	-0.01 [0.00]	-2.98 [4.67]	-0.29 [0.15]	0.02 [0.04]
IPL -> IFG	0.00 [0.00]	-5.45 [4.88]	-0.25 [0.14]	-0.02 [0.03]
ANG -> SMG	-0.02 [0.01]	8.06 [2.21]	-0.11 [0.13]	-0.16 [0.10]*

ANG -> IPL	-0.00 [0.00]	-4.47 [4.86]	-0.28 [0.14]	0.01 [0.02]
ANG -> STG	-0.02 [0.01]	9.05 [2.64]	-0.12 [0.14]	-0.15 [0.01]
ANG -> MTG	0.00 [0.01]	0.75 [4.56]	-0.27 [0.14]	0.00 [0.00]
ANG -> FFG	-0.03 [0.01]	9.47 [2.23]	-0.02 [0.13]	-0.25 [0.10]*
ANG -> IFG	-0.00 [0.00]	1.01 [4.79]	-0.27 [0.14]	0.00 [0.01]
STG -> SMG	-0.03 [0.01]	6.73 [2.43]	-0.09 [0.15]	-0.17 [0.08]*
STG -> IPL	-0.00 [0.00]	4.42 [5.06]	-0.26 [0.14]	-0.01 [0.02]
STG -> ANG	-0.03 [0.01]	7.08 [2.57]	-0.09 [0.15]	-0.18 [0.08]*
STG -> MTG	-0.02 [0.01]	7.84 [2.05]	-0.12 [0.13]	-0.15 [0.08]
STG -> FFG	0.01 [0.00]	0.83 [4.71]	-0.27 [0.14]	0.00 [0.02]
STG -> IFG	0.01 [0.00]	-0.33 [5.35]	-0.27 [0.15]	0.00 [0.04]
MTG -> SMG	0.01 [0.01]	3.10 [4.57]	-0.29 [0.15]	-0.02 [0.03]
MTG -> IPL	-0.02 [0.01]	7.42 [2.16]	-0.12 [0.13]	-0.15 [0.08]
MTG -> ANG	-0.00 [0.00]	-0.69 [4.91]	-0.27 [0.14]	0.00 [0.01]
MTG -> STG	-0.03 [0.01]	4.57 [2.46]	-0.15 [0.15]	-0.12 [0.08]
MTG -> FFG	-0.03 [0.01]	7.52 [2.28]	-0.08 [0.14]	-0.19 [0.08]*
MTG -> IFG	0.00 [0.00]	3.94 [4.83]	-0.28 [0.14]	0.01 [0.02]
FFG -> SMG	-0.00 [0.00]	7.04 [4.86]	-0.25 [0.14]	-0.02 [0.03]
FFG -> IPL	-0.00 [0.00]	-0.41 [5.19]	-0.27 [0.14]	-0.00 [0.00]
FFG -> ANG	-0.02 [0.01]	8.55 [2.18]	-0.06 [0.13]	-0.21 [0.01]*
FFG -> STG	-0.00 [0.00]	-0.91 [4.65]	-0.27 [0.14]	0.00 [0.01]
FFG -> MTG	-0.03 [0.01]	7.64 [2.29]	-0.03 [0.15]	-0.24 [0.09]*
FFG -> IFG	-0.02 [0.01]	10.59 [3.60]	-0.09 [0.14]	-0.18 [0.08]*
IFG -> SMG	-0.00 [0.00]	-1.57 [5.08]	-0.27 [0.14]	-0.01 [0.02]
IFG -> IPL	-0.03 [0.01]	5.34 [2.29]	-0.13 [0.15]	-0.14 [0.08]
IFG -> ANG	-0.00 [0.00]	-8.03 [5.52]	-0.28 [0.14]	-0.02 [0.03]
IFG -> STG	-0.02 [0.01]	7.25 [2.52]	-0.15 [0.14]	-0.12 [0.07]
IFG -> MTG	-0.00 [0.00]	-5.08 [4.90]	-0.27 [0.14]	0.01 [0.02]
IFG -> FFG	-0.02 [0.01]	7.01 [2.36]	-0.11 [0.14]	-0.16 [0.08]*

Supplementary table 4: The table shows all effects (direct and indirect) of mediation analysis for age of intervention (PC1) as predictors, temporal directed partial coherence as a measure for directed functional connectivity as mediators and sentence processing (SEN) subtest of Reading Inventory and Scholastic Evaluation (RISE) as the output measures. The values presented are regression estimates (unstandardized beta values) and standard error. All significant ($p < 0.05$, corrected using BH procedure) indirect effects are highlighted with the red font and asterisks and the corresponding significant direct effects are highlighted with red font.

**RISE – Efficiency of Basic Reading Comprehension (EFFIC)**				
**Connectivity**	**Direct effect: TPDC ~ PC1 (a1)** *Estimate [SE]*	**Direct effect: EFFIC ~ TPDC (b1)** *Estimate [SE]*	**Direct effect: EFFIC ~ PC1 (c1)** *Estimate [SE]*	**Indirect effect: EFFIC ~ PC1 + TPDC (a1*b1)** *Estimate [SE]*
SMG -> IPL	-0.02 [0.01]	6.62 [2.32]	-0.05 [0.14]	-0.16 [0.08]*
SMG -> ANG	-0.01 [0.00]	-0.89 [5.01]	-0.12 [0.15]	-0.01 [0.04]
SMG -> STG	-0.01 [0.00]	-3.16 [5.97]	-0.14 [0.15]	0.02 [0.05]
SMG -> MTG	-0.02 [0.01]	0.66 [2.94]	-0.11 [0.15]	-0.01 [0.05]
SMG -> FFG	-0.00 [0.00]	3.19 [4.92]	-0.10 [0.14]	-0.01 [0.02]
SMG -> IFG	-0.02 [0.01]	4.57 [2.38]	-0.03 [0.15]	-0.08 [0.06]
IPL -> SMG	-0.00 [0.00]	-3.71 [4.76]	-0.11 [0.14]	-0.01 [0.02]

IPL -> ANG	-0.00 [0.01]	-3.06 [4.61]	-0.12 [0.14]	-0.00 [0.02]
IPL -> STG	0.00 [0.00]	3.14 [6.28]	-0.12 [0.14]	0.00 [0.01]
IPL -> MTG	-0.02 [0.01]	2.61 [3.32]	-0.07 [0.15]	-0.04 [0.05]
IPL -> FFG	-0.01 [0.00]	-3.95 [4.70]	-0.14 [0.15]	0.03 [0.04]
IPL -> IFG	0.00 [0.00]	-7.74 [4.87]	-0.09 [0.14]	-0.03 [0.04]
ANG -> SMG	-0.02 [0.01]	5.05 [2.43]	-0.02 [0.15]	-0.09 [0.10]
ANG -> IPL	-0.00 [0.00]	-3.19 [4.93]	-0.12 [0.14]	0.01 [0.02]
ANG -> STG	-0.02 [0.01]	3.51 [2.95]	-0.06 [0.15]	-0.06 [0.05]
ANG -> MTG	0.00 [0.01]	3.11 [4.59]	-0.11 [0.14]	0.00 [0.01]
ANG -> FFG	-0.03 [0.01]	4.43 [2.58]	-0.00 [0.15]	-0.12 [0.07]
ANG -> IFG	-0.00 [0.00]	7.29 [4.72]	-0.12 [0.14]	0.01 [0.03]
STG -> SMG	-0.03 [0.01]	3.43 [2.60]	-0.02 [0.16]	-0.08 [0.07]
STG -> IPL	-0.00 [0.00]	3.85 [5.12]	-0.10 [0.14]	-0.01 [0.02]
STG -> ANG	-0.03 [0.01]	2.22 [2.78]	-0.06 [0.15]	-0.05 [0.07]
STG -> MTG	-0.02 [0.01]	2.98 [2.33]	-0.05 [0.14]	-0.05 [0.05]
STG -> FFG	0.01 [0.00]	1.89 [4.75]	-0.13 [0.15]	0.01 [0.02]
STG -> IFG	-0.01 [0.00]	-5.39 [5.35]	-0.15 [0.15]	0.04 [0.05]
MTG -> SMG	0.01 [0.01]	4.43 [4.59]	-0.15 [0.15]	0.03 [0.04]
MTG -> IPL	-0.02 [0.01]	4.07 [2.37]	-0.03 [0.15]	-0.08 [0.06]
MTG -> ANG	-0.00 [0.00]	5.37 [4.90]	-0.10 [0.14]	-0.01 [0.02]
MTG -> STG	-0.03 [0.01]	2.79 [2.55]	-0.04 [0.15]	-0.07 [0.07]
MTG -> FFG	-0.03 [0.01]	5.19 [2.45]	-0.01 [0.15]	-0.13 [0.07]
MTG -> IFG	0.00 [0.00]	-1.48 [4.91]	-0.11 [0.14]	-0.01 [0.02]
FFG -> SMG	-0.00 [0.00]	4.43 [4.98]	-0.10 [0.14]	-0.01 [0.02]
FFG -> IPL	-0.00 [0.00]	0.39 [5.25]	-0.11 [0.14]	-0.00 [0.00]
FFG -> ANG	-0.02 [0.01]	4.71 [2.45]	-0.00 [0.15]	-0.11 [0.07]
FFG -> STG	-0.00 [0.00]	1.36 [4.70]	-0.11 [0.14]	-0.00 [0.01]
FFG -> MTG	-0.03 [0.01]	4.47 [2.49]	0.02 [0.16]	-0.14 [0.09]
FFG -> IFG	-0.02 [0.01]	7.14 [3.82]	0.00 [0.15]	-0.12 [0.07]
IFG -> SMG	-0.00 [0.00]	-3.04 [5.12]	-0.12 [0.14]	-0.01 [0.02]
IFG -> IPL	-0.03 [0.01]	0.85 [2.45]	-0.09 [0.15]	-0.02 [0.06]
IFG -> ANG	-0.00 [0.00]	-4.71 [5.66]	-0.12 [0.14]	-0.01 [0.02]
IFG -> STG	-0.02 [0.01]	1.95 [2.75]	-0.08 [0.14]	-0.03 [0.04]
IFG -> MTG	-0.00 [0.00]	-0.73 [5.01]	-0.11 [0.14]	-0.00 [0.01]
IFG -> FFG	-0.02 [0.01]	2.62 [2.57]	-0.05 [0.15]	-0.06 [0.06]

Supplementary table 5: The table shows all effects (direct and indirect) of mediation analysis for age of intervention (PC1) as predictors, temporal directed partial coherence as a measure for directed functional connectivity as mediators and efficiency of basic reading comprehension (EFFIC) subtest of Reading Inventory and Scholastic Evaluation (RISE) as the output measures. The values presented are regression estimates (unstandardized beta values) and standard error. All significant ($p < 0.05$, corrected using BH procedure) indirect effects are highlighted with the red font and asterisks and the corresponding significant direct effects are highlighted with red font.

**RISE – Reading Comprehension (RCOMP)**				
**Connectivity**	**Direct effect: RCOMP ~ PC1 (a1)** *Estimate [SE]*	**Direct effect: RCOMP ~ TPDC (b1)** *Estimate [SE]*	**Direct effect: RCOMP ~ PC1 (c1)** *Estimate [SE]*	**Indirect effect: RCOMP ~ PC1 + TPDC (a1*b1)** *Estimate [SE]*
SMG -> IPL	-0.02 [0.01]	7.67 [2.25]	-0.01 [0.14]	-0.19 [0.08]*

SMG -> ANG	-0.01 [0.00]	0.76 [5.02]	-0.19 [0.15]	-0.01 [0.04]
SMG -> STG	-0.01 [0.00]	-1.23 [5.99]	-0.21 [0.15]	0.01 [0.05]
SMG -> MTG	-0.02 [0.01]	2.47 [2.92]	-0.15 [0.15]	-0.04 [0.05]
SMG -> FFG	-0.00 [0.00]	2.66 [4.94]	-0.19 [0.14]	-0.01 [0.02]
SMG -> IFG	-0.02 [0.01]	6.46 [2.29]	-0.08 [0.14]	-0.11 [0.06]
IPL -> SMG	-0.00 [0.00]	-3.60 [4.76]	-0.19 [0.14]	-0.01 [0.02]
IPL -> ANG	-0.00 [0.01]	-1.26 [4.63]	-0.20 [0.14]	-0.00 [0.01]
IPL -> STG	0.00 [0.00]	-7.44 [6.21]	-0.19 [0.14]	-0.00 [0.02]
IPL -> MTG	-0.02 [0.01]	3.32 [3.31]	-0.14 [0.15]	-0.05 [0.05]
IPL -> FFG	-0.01 [0.00]	-2.27 [4.74]	-0.21 [0.15]	0.01 [0.04]
IPL -> IFG	0.00 [0.00]	-10.84 [4.74]	-0.16 [0.14]	-0.04 [0.05]
ANG -> SMG	-0.02 [0.01]	5.09 [2.44]	-0.09 [0.14]	-0.09 [0.06]
ANG -> IPL	-0.00 [0.00]	-0.79 [4.96]	-0.20 [0.14]	0.00 [0.01]
ANG -> STG	-0.02 [0.01]	4.97 [2.90]	-0.12 [0.15]	-0.08 [0.06]
ANG -> MTG	0.00 [0.01]	-0.26 [4.62]	-0.19 [0.14]	-0.00 [0.01]
ANG -> FFG	-0.03 [0.01]	5.31 [2.54]	-0.06 [0.15]	-0.14 [0.08]
ANG -> IFG	-0.00 [0.00]	4.35 [4.81]	-0.20 [0.14]	-0.00 [0.02]
STG -> SMG	-0.03 [0.01]	4.22 [2.58]	-0.08 [0.15]	-0.11 [0.07]
STG -> IPL	-0.00 [0.00]	0.85 [5.16]	-0.19 [0.14]	-0.00 [0.01]
STG -> ANG	-0.03 [0.01]	3.25 [2.76]	-0.12 [0.15]	-0.08 [0.07]
STG -> MTG	-0.02 [0.01]	4.10 [2.30]	-0.11 [0.14]	-0.08 [0.05]
STG -> FFG	0.01 [0.00]	2.62 [4.75]	-0.21 [0.14]	0.01 [0.02]
STG -> IFG	-0.01 [0.00]	-8.02 [5.23]	-0.26 [0.15]	0.06 [0.05]
MTG -> SMG	0.01 [0.01]	4.40 [4.60]	-0.22 [0.14]	0.03 [0.04]
MTG -> IPL	-0.02 [0.01]	5.27 [2.32]	-0.09 [0.14]	-0.10 [0.06]
MTG -> ANG	-0.00 [0.00]	0.64 [4.97]	-0.19 [0.14]	-0.00 [0.01]
MTG -> STG	-0.03 [0.01]	2.67 [2.55]	-0.12 [0.15]	-0.07 [0.07]
MTG -> FFG	-0.03 [0.01]	5.85 [2.42]	-0.05 [0.15]	-0.15 [0.07]
MTG -> IFG	0.00 [0.00]	-1.10 [4.92]	-0.19 [0.14]	-0.00 [0.02]
FFG -> SMG	-0.00 [0.00]	5.10 [4.97]	-0.18 [0.14]	-0.01 [0.02]
FFG -> IPL	-0.00 [0.00]	4.95 [5.20]	-0.19 [0.14]	-0.00 [0.02]
FFG -> ANG	-0.02 [0.01]	5.38 [2.42]	-0.06 [0.15]	-0.13 [0.07]
FFG -> STG	-0.00 [0.00]	-0.89 [4.71]	-0.19 [0.14]	0.00 [0.01]
FFG -> MTG	-0.03 [0.01]	4.80 [2.48]	-0.04 [0.15]	-0.15 [0.08]
FFG -> IFG	-0.02 [0.01]	7.18 [3.82]	-0.07 [0.15]	-0.12 [0.07]
IFG -> SMG	-0.00 [0.00]	-4.16 [5.11]	-0.21 [0.14]	-0.02 [0.03]
IFG -> IPL	-0.03 [0.01]	2.29 [2.43]	-0.13 [0.15]	-0.06 [0.07]
IFG -> ANG	-0.00 [0.00]	-0.47 [5.71]	-0.19 [0.14]	0.00 [0.01]
IFG -> STG	-0.02 [0.01]	4.07 [2.70]	-0.13 [0.13]	-0.06 [0.05]
IFG -> MTG	-0.00 [0.00]	-7.23 [4.90]	-0.20 [0.14]	-0.01 [0.03]
IFG -> FFG	-0.02 [0.01]	4.38 [2.53]	-0.10 [0.15]	-0.09 [0.06]

Supplementary table 6: The table shows all effects (direct and indirect) of mediation analysis for age of intervention (PC1) as predictors, temporal directed partial coherence as a measure for directed functional connectivity as mediators and reading comprehension (RCOMP) subtest of Reading Inventory and Scholastic Evaluation (RISE) as the output measures. The values presented are regression estimates (unstandardized beta values) and standard error. All significant ($p < 0.05$) indirect effects are highlighted with the red font and asterisks and the corresponding significant direct effects are highlighted with red font.

<u>Mediation analysis with PC3 and each subtest of RISE for children with CI</u>

RISE - Word Recognition and Decoding (WRDC)				
**Connectivity**	**Direct effect: TPDC ~ PC3 (a1)** *Estimate [SE]*	**Direct effect: WRDC ~ TPDC (b1)** *Estimate [SE]*	**Direct effect: WRDC ~ PC3 (c1)** *Estimate [SE]*	**Indirect effect: WRDC ~ PC3 + TPDC (c1′)** *Estimate [SE]*
SMG -> IPL	-0.02 [0.01]	12.31 [1.54]	0.34 [0.01]	-0.18 [0.11]
SMG -> ANG	-0.01 [0.00]	17.43 [4.64]	0.38 [0.14]	-0.22 [0.09]*
SMG -> STG	-0.01 [0.00]	-1.58 [5.92]	0.15 [0.15]	0.01 [0.04]
SMG -> MTG	-0.03 [0.01]	11.5 [2.67]	0.45 [0.14]	-0.29 [0.00]*
SMG -> FFG	-0.01 [0.00]	4.64 [4.94]	0.18 [0.14]	-0.02 [0.03]
SMG -> IFG	-0.02 [0.01]	12.12 [1.84]	0.45 [0.11]	-0.29 [0.11]*
IPL -> SMG	0.01 [0.00]	-4.96 [4.80]	0.19 [0.14]	-0.03 [0.04]
IPL -> ANG	0.00 [0.01]	2.92 [4.61]	0.16 [0.14]	0.00 [0.01]
IPL -> STG	-0.00 [0.00]	-4.10 [6.26]	0.16 [0.14]	0.00 [0.01]
IPL -> MTG	-0.02 [0.01]	14.57 [2.99]	0.50 [0.14]	-0.34 [0.11]*
IPL -> FFG	-0.00 [0.01]	-1.98 [4.64]	0.15 [0.14]	0.01 [0.02]
IPL -> IFG	-0.01 [0.00]	-12.45 [4.69]	0.09 [0.13]	-0.06 [0.05]
ANG -> SMG	-0.02 [0.01]	10.94 [1.91]	0.34 [0.11]	-0.18 [0.10]
ANG -> IPL	0.00 [0.00]	-3.71 [4.89]	0.16 [0.14]	-0.00 [0.02]
ANG -> STG	-0.02 [0.01]	9.76 [2.59]	0.30 [0.13]	-0.14 [0.01]
ANG -> MTG	0.00 [0.01]	2.64 [4.61]	0.15 [0.14]	0.01 [0.02]
ANG -> FFG	-0.02 [0.01]	12.56 [1.71]	0.39 [0.10]	-0.24 [0.11]*
ANG -> IFG	-0.00 [0.00]	-1.30 [4.84]	0.16 [0.14]	-0.00 [0.01]
STG -> SMG	-0.02 [0.01]	10.15 [1.96]	0.31 [0.12]	-0.15 [0.09]
STG -> IPL	-0.00 [0.00]	5.59 [5.09]	0.15 [0.14]	0.01 [0.03]
STG -> ANG	-0.02 [0.01]	10.35 [2.26]	0.38 [0.13]	-0.23 [0.09]*
STG -> MTG	-0.03 [0.01]	12.11 [1.68]	0.45 [0.11]	-0.30 [0.11]*
STG -> FFG	0.01 [0.00]	-6.18 [4.83]	0.21 [0.15]	-0.05 [0.05]
STG -> IFG	-0.00 [0.00]	0.73 [5.24]	0.16 [0.15]	-0.00 [0.02]
MTG -> SMG	0.00 [0.01]	-3.59 [4.52]	0.17 [0.14]	-0.01 [0.02]
MTG -> IPL	-0.02 [0.01]	11.51 [1.76]	0.39 [0.11]	-0.24 [0.11]*
MTG -> ANG	-0.00 [0.00]	-2.23 [4.94]	0.16 [0.14]	-0.00 [0.01]
MTG -> STG	-0.03 [0.01]	9.48 [2.14]	0.40 [0.13]	-0.25 [0.09]*
MTG -> FFG	-0.03 [0.01]	12.43 [1.81]	0.47 [0.11]	-0.31 [0.11]*
MTG -> IFG	0.00 [0.00]	0.65 [4.93]	0.15 [0.14]	0.00 [0.02]
FFG -> SMG	-0.00 [0.00]	6.17 [4.92]	0.17 [0.14]	-0.01 [0.03]
FFG -> IPL	0.00 [0.00]	5.18 [5.24]	0.14 [0.14]	0.02 [0.03]
FFG -> ANG	-0.02 [0.01]	11.15 [1.79]	0.35 [0.11]	-0.19 [0.10]
FFG -> STG	-0.01 [0.00]	-0.82 [4.76]	0.15 [0.14]	0.00 [0.02]
FFG -> MTG	-0.03 [0.01]	11.22 [1.77]	0.43 [0.11]	-0.28 [0.11]*
FFG -> IFG	-0.01 [0.01]	17.56 [2.65]	0.33 [0.10]	-0.17 [0.09]
IFG -> SMG	-0.00 [0.00]	-1.70 [5.36]	0.14 [0.15]	-0.02 [0.05]
IFG -> IPL	-0.03 [0.01]	10.02 [1.92]	0.41 [0.12]	-0.25 [0.10]*
IFG -> ANG	0.01 [0.00]	-5.81 [5.84]	0.19 [0.14]	-0.04 [0.05]
IFG -> STG	-0.01 [0.01]	11.10 [2.18]	0.31 [0.12]	-0.16 [0.09]
IFG -> MTG	-0.00 [0.00]	-3.76 [5.01]	0.17 [0.14]	-0.01 [0.02]
IFG -> FFG	-0.02 [0.01]	10.51 [2.07]	0.38 [0.12]	-0.23 [0.09]*

Supplementary table 7: The table shows all effects (direct and indirect) of mediation analysis for developmental factor (PC3) as predictors, temporal directed partial coherence as a measure for

directed functional connectivity as mediators and word recognition and decoding (WRDC) subtest of Reading Inventory and Scholastic Evaluation (RISE) as the output measures. The values presented are regression estimates (unstandardized beta values) and standard error. All significant ($p < 0.05$, corrected using BH procedure) indirect effects are highlighted with the red font and asterisks and the corresponding significant direct effects are highlighted with red font.

**RISE – Vocabulary (VOC)**				
**Connectivity**	**Direct effect: TPDC ~ PC3 (a1)** *Estimate [SE]*	**Direct effect: VOC ~ TPDC (b1)** *Estimate [SE]*	**Direct effect: VOC ~ PC3 (c1)** *Estimate [SE]*	**Indirect effect: VOC ~ PC3 + TPDC (a1*b1)** *Estimate [SE]*
SMG -> IPL	-0.02 [0.01]	10.97 [1.58]	0.51 [0.09]	-0.16 [0.10]
SMG -> ANG	-0.01 [0.00]	10.85 [4.78]	0.48 [0.14]	-0.14 [0.07]
SMG -> STG	-0.01 [0.00]	-0.74 [5.63]	0.34 [0.14]	0.01 [0.04]
SMG -> MTG	-0.03 [0.01]	9.78 [2.64]	0.59 [0.14]	-0.24 [0.09]*
SMG -> FFG	-0.01 [0.00]	5.87 [4.65]	0.38 [0.14]	-0.03 [0.03]
SMG -> IFG	-0.02 [0.01]	11.37 [1.77]	0.62 [0.11]	-0.27 [0.10]*
IPL -> SMG	0.01 [0.00]	-4.58 [4.56]	0.37 [0.14]	-0.03 [0.03]
IPL -> ANG	0.00 [0.01]	-2.94 [4.37]	0.35 [0.14]	-0.00 [0.01]
IPL -> STG	-0.00 [0.00]	0.39 [5.97]	0.35 [0.14]	-0.00 [0.00]
IPL -> MTG	-0.02 [0.01]	12.43 [2.98]	0.64 [0.14]	-0.29 [0.10]*
IPL -> FFG	-0.00 [0.01]	-1.68 [4.41]	0.34 [0.14]	0.01 [0.02]
IPL -> IFG	-0.01 [0.00]	-10.47 [4.53]	0.29 [0.13]	0.05 [0.05]
ANG -> SMG	-0.02 [0.01]	9.47 [1.92]	0.51 [0.12]	-0.16 [0.10]
ANG -> IPL	-0.00 [0.00]	1.44 [4.67]	0.35 [0.14]	0.00 [0.01]
ANG -> STG	-0.02 [0.01]	9.54 [2.44]	0.49 [0.12]	-0.14 [0.07]
ANG -> MTG	0.00 [0.01]	0.93 [4.39]	0.35 [0.14]	0.00 [0.01]
ANG -> FFG	-0.02 [0.01]	10.55 [1.82]	0.55 [0.11]	-0.20 [0.09]*
ANG -> IFG	0.00 [0.00]	-0.08 [4.60]	0.35 [0.14]	-0.00 [0.01]
STG -> SMG	-0.02 [0.01]	8.62 [1.97]	0.47 [0.12]	-0.13 [0.08]
STG -> IPL	0.00 [0.00]	2.91 [4.88]	0.34 [0.14]	0.01 [0.02]
STG -> ANG	-0.02 [0.01]	7.68 [2.34]	0.52 [0.13]	-0.17 [0.08]*
STG -> MTG	-0.03 [0.01]	9.83 [1.82]	0.59 [0.12]	-0.24 [0.09]*
STG -> FFG	0.01 [0.00]	-3.54 [4.64]	0.38 [0.14]	-0.03 [0.04]
STG -> IFG	-0.00 [0.00]	-3.44 [4.96]	0.34 [0.14]	0.01 [0.02]
MTG -> SMG	0.00 [0.01]	-0.59 [4.32]	0.35 [0.14]	-0.00 [0.02]
MTG -> IPL	-0.02 [0.01]	10.63 [1.72]	0.57 [0.11]	-0.22 [0.09]*
MTG -> ANG	0.00 [0.00]	-0.08 [4.70]	0.35 [0.14]	-0.00 [0.01]
MTG -> STG	-0.03 [0.01]	7.69 [2.15]	0.55 [0.13]	-0.20 [0.08]*
MTG -> FFG	-0.03 [0.01]	10.62 [1.88]	0.62 [0.12]	-0.27 [0.09]*
MTG -> IFG	0.00 [0.00]	-1.89 [4.67]	0.36 [0.14]	-0.01 [0.02]
FFG -> SMG	-0.00 [0.00]	7.14 [4.64]	0.37 [0.13]	-0.02 [0.03]
FFG -> IPL	0.00 [0.00]	2.79 [5.01]	0.34 [0.14]	0.01 [0.02]
FFG -> ANG	-0.02 [0.01]	9.49 [1.84]	0.51 [0.11]	-0.17 [0.08]
FFG -> STG	-0.01 [0.00]	-0.06 [4.53]	0.35 [0.14]	0.00 [0.02]
FFG -> MTG	-0.03 [0.01]	9.62 [1.81]	0.58 [0.12]	-0.24 [0.09]*
FFG -> IFG	-0.01 [0.01]	14.74 [2.76]	0.49 [0.11]	-0.14 [0.08]
IFG -> SMG	-0.01 [0.00]	-1.31 [5.09]	0.34 [0.14]	0.01 [0.05]
IFG -> IPL	-0.03 [0.01]	7.68 [2.00]	0.54 [0.13]	-0.19 [0.08]*
IFG -> ANG	0.01 [0.00]	-2.49 [5.59]	0.37 [0.14]	-0.02 [0.04]
IFG -> STG	-0.01 [0.01]	9.49 [2.19]	0.48 [0.12]	-0.13 [0.08]

IFG -> MTG	0.00 [0.00]	-5.73 [4.71]	0.37 [0.14]	-0.02 [0.03]
IFG -> FFG	-0.02 [0.01]	9.58 [2.02]	0.56 [0.12]	-0.21 [0.09]*

Supplementary table 8: The table shows all effects (direct and indirect) of mediation analysis for developmental factor (PC3) as predictors, temporal directed partial coherence as a measure for directed functional connectivity as mediators and word recognition and decoding (VOC) subtest of Reading Inventory and Scholastic Evaluation (RISE) as the output measures. The values presented are regression estimates (unstandardized beta values) and standard error. All significant ($p < 0.05$, corrected using BH procedure) indirect effects are highlighted with the red font and asterisks and the corresponding significant direct effects are highlighted with red font.

**RISE – Morphology (MORPH)**				
**Connectivity**	**Direct effect: TPDC ~ PC3 (a1)** *Estimate [SE]*	**Direct effect: MORPH ~ TPDC (b1)** *Estimate [SE]*	**Direct effect: MORPH ~ PC3 (c1)** *Estimate [SE]*	**Indirect effect: MORPH ~ PC3 + TPDC (a1*b1)** *Estimate [SE]*
SMG -> IPL	-0.02 [0.01]	11.07 [1.64]	0.47 [0.10]	-0.16 [0.10]
SMG -> ANG	-0.01 [0.00]	12.39 [4.82]	0.46 [0.14]	-0.16 [0.08]*
SMG -> STG	-0.01 [0.00]	0.03 [5.75]	0.31 [0.15]	-0.00 [0.04]
SMG -> MTG	-0.03 [0.01]	11.63 [2.55]	0.60 [0.13]	-0.29 [0.10]*
SMG -> FFG	-0.01 [0.00]	2.80 [4.82]	0.32 [0.14]	-0.01 [0.03]
SMG -> IFG	-0.02 [0.01]	12.07 [1.74]	0.59 [0.11]	-0.29 [0.11]*
IPL -> SMG	0.01 [0.00]	-4.93 [4.66]	0.34 [0.14]	-0.03 [0.03]
IPL -> ANG	0.00 [0.01]	-2.96 [4.47]	0.31 [0.14]	-0.00 [0.01]
IPL -> STG	-0.00 [0.00]	-6.74 [6.03]	0.31 [0.14]	-0.00 [0.02]
IPL -> MTG	-0.02 [0.01]	15.09 [2.79]	0.67 [0.13]	-0.36 [0.11]*
IPL -> FFG	-0.01 [0.01]	-2.21 [4.50]	0.30 [0.14]	0.01 [0.02]
IPL -> IFG	-0.01 [0.00]	-11.18 [4.60]	0.26 [0.13]	0.05 [0.05]
ANG -> SMG	-0.02 [0.01]	10.37 [1.88]	0.48 [0.11]	-0.17 [0.09]
ANG -> IPL	0.00 [0.00]	-3.86 [4.75]	0.31 [0.14]	-0.00 [0.02]
ANG -> STG	-0.02 [0.01]	10.25 [2.45]	0.46 [0.12]	-0.15 [0.08]
ANG -> MTG	0.00 [0.01]	1.23 [4.48]	0.31 [0.14]	0.00 [0.01]
ANG -> FFG	-0.02 [0.01]	11.46 [1.76]	0.52 [0.11]	-0.22 [0.10]*
ANG -> IFG	0.00 [0.00]	-0.93 [4.70]	0.31 [0.14]	-0.00 [0.00]
STG -> SMG	-0.02 [0.01]	8.76 [2.01]	0.44 [0.12]	-0.13 [0.08]
STG -> IPL	0.00 [0.00]	3.14 [4.98]	0.30 [0.14]	0.01 [0.02]
STG -> ANG	-0.02 [0.01]	9.17 [2.28]	0.51 [0.13]	-0.20 [0.09]*
STG -> MTG	-0.03 [0.01]	10.90 [1.75]	0.58 [0.11]	-0.27 [0.10]*
STG -> FFG	0.01 [0.00]	-3.98 [4.73]	0.35 [0.14]	-0.04 [0.05]
STG -> IFG	-0.00 [0.00]	-1.07 [5.09]	0.31 [0.14]	0.00 [0.02]
MTG -> SMG	0.00 [0.01]	-1.15 [4.42]	0.31 [0.14]	-0.00 [0.02]
MTG -> IPL	-0.02 [0.01]	11.29 [1.70]	0.54 [0.11]	-0.23 [0.10]*
MTG -> ANG	0.00 [0.00]	2.08 [4.79]	0.31 [0.14]	0.00 [0.01]
MTG -> STG	-0.03 [0.01]	9.40 [2.06]	0.55 [0.13]	-0.24 [0.09]*
MTG -> FFG	-0.03 [0.01]	11.92 [1.77]	0.61 [0.11]	-0.30 [0.11]*
MTG -> IFG	0.00 [0.00]	0.07 [4.78]	0.31 [0.14]	0.00 [0.02]
FFG -> SMG	-0.00 [0.00]	2.42 [4.85]	0.32 [0.14]	-0.01 [0.02]
FFG -> IPL	0.00 [0.00]	-0.56 [5.14]	0.31 [0.14]	-0.00 [0.00]
FFG -> ANG	-0.02 [0.01]	10.36 [1.79]	0.48 [0.11]	-0.17 [0.09]
FFG -> STG	-0.01 [0.00]	1.55 [4.62]	0.32 [0.14]	-0.01 [0.02]

FFG -> MTG	-0.03 [0.01]	10.67 [1.75]	0.57 [0.11]	-0.26 [0.10]*
FFG -> IFG	-0.01 [0.01]	14.38 [2.90]	0.45 [0.11]	-0.14 [0.09]
IFG -> SMG	-0.01 [0.00]	1.35 [5.21]	0.32 [0.15]	-0.01 [0.05]
IFG -> IPL	-0.03 [0.01]	8.85 [1.96]	0.53 [0.13]	-0.22 [0.09]*
IFG -> ANG	0.01 [0.00]	-8.03 [5.61]	0.37 [0.14]	-0.05 [0.05]
IFG -> STG	-0.01 [0.01]	9.39 [2.26]	0.44 [0.12]	-0.13 [0.08]
IFG -> MTG	0.00 [0.00]	-9.67 [4.68]	0.34 [0.13]	-0.03 [0.04]
IFG -> FFG	-0.02 [0.01]	10.06 [2.03]	0.53 [0.12]	-0.22 [0.09]*

Supplementary table 9: The table shows all effects (direct and indirect) of mediation analysis for developmental factor (PC3) as predictors, temporal directed partial coherence as a measure for directed functional connectivity as mediators and morphology (MORPH) subtest of Reading Inventory and Scholastic Evaluation (RISE) as the output measures. The values presented are regression estimates (unstandardized beta values) and standard error. All significant ($p < 0.05$, corrected using BH procedure) indirect effects are highlighted with the red font and asterisks and the corresponding significant direct effects are highlighted with red font.

**RISE – Sentence Processing (SEN)**				
**Connectivity**	**Direct effect: TPDC ~ PC3 (a1)** *Estimate [SE]*	**Direct effect: SEN ~ TPDC (b1)** *Estimate [SE]*	**Direct effect: SEN ~ PC3 (c1)** *Estimate [SE]*	**Indirect effect: SEN ~ PC3 + TPDC (a1*b1)** *Estimate [SE]*
SMG -> IPL	-0.02 [0.01]	11.56 [1.75]	0.25 [0.11]	-0.17 [0.11]
SMG -> ANG	-0.01 [0.00]	18.69 [4.68]	0.32 [0.14]	-0.24 [0.09]*
SMG -> STG	-0.01 [0.00]	1.83 [6.06]	0.09 [0.15]	-0.01 [0.04]
SMG -> MTG	-0.03 [0.01]	10.42 [2.86]	0.34 [0.15]	-0.26 [0.10]*
SMG -> FFG	-0.01 [0.00]	3.27 [5.08]	0.09 [0.15]	-0.02 [0.03]
SMG -> IFG	-0.02 [0.01]	12.06 [1.94]	0.37 [0.12]	-0.29 [0.11]*
IPL -> SMG	0.01 [0.00]	-0.78 [4.97]	0.09 [0.15]	-0.00 [0.03]
IPL -> ANG	0.00 [0.01]	1.41 [4.74]	0.08 [0.15]	-0.00 [0.01]
IPL -> STG	-0.00 [0.00]	-4.54 [6.41]	0.08 [0.15]	0.00 [0.02]
IPL -> MTG	-0.02 [0.01]	12.36 [3.30]	0.37 [0.15]	-0.29 [0.11]*
IPL -> FFG	-0.00 [0.01]	-0.55 [4.76]	0.07 [0.15]	0.00 [0.02]
IPL -> IFG	-0.01 [0.00]	-6.21 [5.07]	0.05 [0.15]	0.03 [0.04]
ANG -> SMG	-0.02 [0.01]	9.81 [2.11]	0.24 [0.13]	-0.16 [0.10]
ANG -> IPL	-0.00 [0.00]	-3.53 [5.02]	0.08 [0.15]	-0.00 [0.02]
ANG -> STG	-0.02 [0.01]	11.24 [2.54]	0.25 [0.13]	-0.17 [0.09]
ANG -> MTG	0.00 [0.01]	0.55 [4.74]	0.08 [0.15]	0.00 [0.01]
ANG -> FFG	-0.02 [0.01]	11.15 [1.99]	0.29 [0.12]	-0.21 [0.10]*
ANG -> IFG	0.00 [0.00]	0.63 [4.96]	0.08 [0.15]	0.00 [0.01]
STG -> SMG	-0.02 [0.13]	8.23 [2.22]	0.20 [0.13]	-0.12 [0.08]
STG -> IPL	0.00 [0.00]	4.91 [5.23]	0.07 [0.15]	0.01 [0.02]
STG -> ANG	-0.02 [0.01]	9.79 [2.39]	0.29 [0.14]	-0.22 [0.09]*
STG -> MTG	-0.03 [0.01]	10.45 [1.98]	0.34 [0.13]	-0.26 [0.10]*
STG -> FFG	0.01 [0.00]	-1.41 [5.03]	0.09 [0.15]	-0.01 [0.04]
STG -> IFG	-0.00 [0.00]	2.84 [5.36]	0.09 [0.15]	-0.01 [0.02]
MTG -> SMG	0.00 [0.01]	0.88 [4.66]	0.08 [0.15]	0.00 [0.02]
MTG -> IPL	-0.02 [0.01]	9.51 [2.08]	0.27 [0.13]	-0.19 [0.09]*
MTG -> ANG	0.00 [0.00]	-0.04 [5.07]	0.08 [0.15]	-0.00 [0.01]
MTG -> STG	-0.03 [0.01]	7.44 [2.27]	0.27 [0.15]	-0.19 [0.09]*

MTG -> FFG	-0.03 [0.01]	10.37 [2.15]	0.34 [0.13]	-0.26 [0.10]*
MTG -> IFG	0.00 [0.00]	2.59 [5.03]	0.07 [0.15]	0.01 [0.02]
FFG -> SMG	-0.00 [0.00]	8.19 [4.98]	0.10 [0.14]	-0.02 [0.04]
FFG -> IPL	0.00 [0.00]	-0.77 [5.42]	0.08 [0.15]	-0.00 [0.02]
FFG -> ANG	-0.02 [0.01]	10.06 [1.99]	0.25 [0.12]	-0.17 [0.09]
FFG -> STG	-0.01 [0.00]	-0.22 [4.88]	0.08 [0.15]	0.00 [0.02]
FFG -> MTG	-0.03 [0.01]	9.75 [2.02]	0.32 [0.13]	-0.24 [0.09]*
FFG -> IFG	-0.01 [0.01]	12.79 [3.29]	0.21 [0.13]	-0.13 [0.08]
IFG -> SMG	-0.01 [0.00]	0.85 [5.50]	0.09 [0.15]	-0.01 [0.05]
IFG -> IPL	-0.03 [0.01]	7.81 [2.20]	0.28 [0.14]	-0.19 [0.09]*
IFG -> ANG	0.01 [0.00]	-8.74 [5.91]	0.14 [0.15]	-0.06 [0.05]
IFG -> STG	-0.01 [0.01]	9.06 [2.46]	0.21 [0.13]	-0.13 [0.08]
IFG -> MTG	0.00 [0.00]	-4.99 [5.11]	0.09 [0.14]	-0.02 [0.02]
IFG -> FFG	-0.02 [0.01]	9.47 [2.26]	0.29 [0.13]	-0.21 [0.09]*

Supplementary table 10: The table shows all effects (direct and indirect) of mediation analysis for developmental factor (PC3) as predictors, temporal directed partial coherence as a measure for directed functional connectivity as mediators and sentence processing (SEN) subtest of Reading Inventory and Scholastic Evaluation (RISE) as the output measures. The values presented are regression estimates (unstandardized beta values) and standard error. All significant ($p < 0.05$, corrected using BH procedure) indirect effects are highlighted with the red font and asterisks and the corresponding significant direct effects are highlighted with red font.

**RISE – Efficiency of Basic Reading Comprehension (EFFIC)**				
**Connectivity**	**Direct effect: TPDC ~ PC3 (a1)** *Estimate [SE]*	**Direct effect: EFFIC ~ TPDC (b1)** *Estimate [SE]*	**Direct effect: EFFIC ~ PC3 (c1)** *Estimate [SE]*	**Indirect effect: EFFIC ~ PC3 + TPDC (a1*b1)** *Estimate [SE]*
SMG -> IPL	-0.02 [0.01]	8.12 [1.90]	0.47 [0.12]	-0.12 [0.08]
SMG -> ANG	-0.01 [0.00]	6.37 [4.92]	0.43 [0.15]	-0.08 [0.07]
SMG -> STG	-0.01 [0.00]	2.59 [5.57]	0.37 [0.14]	-0.02 [0.04]
SMG -> MTG	-0.03 [0.01]	5.95 [2.85]	0.49 [0.15]	-0.15 [0.08]
SMG -> FFG	-0.01 [0.00]	5.53 [4.63]	0.37 [0.14]	-0.03 [0.03]
SMG -> IFG	-0.02 [0.01]	8.22 [2.09]	0.54 [0.13]	-0.20 [0.08]*
IPL -> SMG	0.01 [0.00]	-6.38 [4.48]	0.38 [0.14]	-0.04 [0.04]
IPL -> ANG	0.00 [0.01]	-3.27 [4.34]	0.35 [0.14]	-0.00 [0.02]
IPL -> STG	-0.00 [0.00]	3.25 [5.91]	0.35 [0.14]	-0.00 [0.01]
IPL -> MTG	-0.02 [0.01]	9.70 [3.16]	0.57 [0.14]	-0.23 [0.09]*
IPL -> FFG	-0.01 [0.01]	-1.57 [4.37]	0.34 [0.14]	0.01 [0.02]
IPL -> IFG	-0.01 [0.00]	-6.27 [4.65]	0.32 [0.14]	0.03 [0.04]
ANG -> SMG	-0.02 [0.01]	7.34 [2.09]	0.47 [0.13]	-0.12 [0.07]
ANG -> IPL	0.00 [0.00]	-2.98 [4.62]	0.35 [0.14]	-0.00 [0.01]
ANG -> STG	-0.02 [0.01]	6.44 [2.63]	0.44 [0.13]	-0.09 [0.06]
ANG -> MTG	0.00 [0.01]	2.23 [4.34]	0.34 [0.14]	0.01 [0.02]
ANG -> FFG	-0.02 [0.01]	6.95 [2.15]	0.48 [0.13]	-0.13 [0.07]
ANG -> IFG	0.00 [0.00]	6.63 [4.46]	0.34 [0.13]	0.01 [0.03]
STG -> SMG	-0.02 [0.01]	5.38 [2.18]	0.47 [0.13]	-0.08 [0.06]
STG -> IPL	0.00 [0.00]	3.14 [4.84]	0.34 [0.14]	0.01 [0.02]
STG -> ANG	-0.02 [0.01]	5.92 [2.42]	0.47 [0.14]	-0.13 [0.07]
STG -> MTG	-0.02 [0.01]	6.32 [2.11]	0.50 [0.13]	-0.15 [0.07]*

STG -> FFG	0.01 [0.00]	-2.19 [4.62]	0.37 [0.14]	-0.02 [0.04]
STG -> IFG	-0.00 [0.00]	-2.07 [4.94]	0.34 [0.14]	0.01 [0.02]
MTG -> SMG	0.00 [0.01]	2.31 [4.28]	0.34 [0.14]	0.01 [0.02]
MTG -> IPL	-0.02 [0.01]	6.84 [2.08]	0.48 [0.13]	-0.14 [0.07]
MTG -> ANG	0.00 [0.00]	4.97 [4.61]	0.34 [0.13]	0.01 [0.02]
MTG -> STG	-0.03 [0.01]	6.59 [2.21]	0.52 [0.14]	-0.17 [0.07]*
MTG -> FFG	-0.03 [0.01]	8.97 [2.03]	0.57 [0.12]	-0.23 [0.09]*
MTG -> IFG	0.00 [0.00]	-3.53 [4.62]	0.36 [0.14]	-0.01 [0.02]
FFG -> SMG	-0.00 [0.00]	5.81 [4.64]	0.36 [0.13]	-0.01 [0.03]
FFG -> IPL	0.00 [0.00]	-1.42 [4.98]	0.35 [0.14]	-0.01 [0.02]
FFG -> ANG	-0.02 [0.01]	6.79 [2.06]	0.46 [0.13]	-0.11 [0.07]
FFG -> STG	-0.00 [0.00]	3.37 [4.47]	0.36 [0.14]	-0.02 [0.03]
FFG -> MTG	-0.03 [0.01]	7.44 [2.00]	0.53 [0.13]	-0.18 [0.08]*
FFG -> IFG	-0.01 [0.01]	9.81 [3.18]	0.44 [0.13]	-0.10 [0.06]
IFG -> SMG	-0.00 [0.00]	1.76 [5.06]	0.36 [0.14]	-0.02 [0.04]
IFG -> IPL	-0.03 [0.01]	4.17 [2.20]	0.45 [0.14]	-0.10 [0.07]
IFG -> ANG	0.01 [0.00]	-8.75 [5.42]	0.41 [0.14]	-0.06 [0.05]
IFG -> STG	-0.01 [0.01]	4.36 [2.49]	0.41 [0.14]	-0.06 [0.05]
IFG -> MTG	0.00 [0.00]	-2.00 [4.74]	0.35 [0.14]	-0.01 [0.02]
IFG -> FFG	-0.02 [0.01]	5.91 [2.28]	0.47 [0.13]	-0.13 [0.06]

Supplementary table 11: The table shows all effects (direct and indirect) of mediation analysis for developmental factor (PC3) as predictors, temporal directed partial coherence as a measure for directed functional connectivity as mediators and efficiency of basic reading comprehension (EFFIC) subtest of Reading Inventory and Scholastic Evaluation (RISE) as the output measures. The values presented are regression estimates (unstandardized beta values) and standard error. All significant ($p < 0.05$, corrected using BH procedure) indirect effects are highlighted with the red font and asterisks and the corresponding significant direct effects are highlighted with red font.

**RISE – Reading Comprehension (RCOMP)**				
**Connectivity**	**Direct effect: RCOMP ~ PC3 (a1)** *Estimate [SE]*	**Direct effect: RCOMP ~ TPDC (b1)** *Estimate [SE]*	**Direct effect: RCOMP ~ PC3 (c1)** *Estimate [SE]*	**Indirect effect: RCOMP ~ PC3 + TPDC (a1*b1)** *Estimate [SE]*
SMG -> IPL	-0.02 [0.01]	9.58 [1.81]	0.48 [0.11]	-0.14 [0.08]
SMG -> ANG	-0.01 [0.00]	9.04 [4.93]	0.45 [0.15]	-0.12 [0.07]
SMG -> STG	-0.01 [0.00]	5.46 [5.64]	0.37 [0.14]	-0.04 [0.04]
SMG -> MTG	-0.03 [0.01]	8.65 [2.77]	0.55 [0.14]	-0.22 [0.09]*
SMG -> FFG	-0.00 [0.00]	5.15 [4.74]	0.36 [0.14]	-0.02 [0.03]
SMG -> IFG	-0.02 [0.01]	10.66 [1.90]	0.59 [0.12]	-0.25 [0.09]*
IPL -> SMG	0.01 [0.00]	-6.45 [4.57]	0.37 [0.14]	-0.04 [0.04]
IPL -> ANG	0.00 [0.01]	-1.34 [4.45]	0.34 [0.14]	-0.00 [0.01]
IPL -> STG	-0.00 [0.00]	-7.34 [5.95]	0.34 [0.14]	0.00 [0.03]
IPL -> MTG	-0.02 [0.01]	11.25 [3.12]	0.60 [0.14]	-0.26 [0.09]*
IPL -> FFG	-0.01 [0.01]	0.62 [4.47]	0.34 [0.14]	-0.00 [0.02]
IPL -> IFG	-0.01 [0.00]	-9.82 [4.62]	0.29 [0.13]	0.05 [0.05]
ANG -> SMG	-0.02 [0.01]	7.82 [2.11]	0.47 [0.13]	-0.13 [0.07]
ANG -> IPL	0.00 [0.00]	-0.32 [4.74]	0.34 [0.14]	-0.00 [0.00]
ANG -> STG	-0.02 [0.01]	8.38 [2.57]	0.46 [0.13]	-0.12 [0.07]
ANG -> MTG	0.00 [0.01]	-1.15 [4.44]	0.34 [0.14]	-0.00 [0.01]

ANG -> FFG	-0.02 [0.01]	8.35 [2.09]	0.49 [0.13]	-0.16 [0.08]*
ANG -> IFG	0.00 [0.00]	3.63 [4.63]	0.33 [0.14]	0.01 [0.02]
STG -> SMG	-0.02 [0.01]	6.66 [2.16]	0.44 [0.13]	-0.09 [0.07]
STG -> IPL	0.00 [0.00]	0.39 [4.96]	0.34 [0.14]	0.00 [0.01]
STG -> ANG	-0.02 [0.01]	7.58 [2.38]	0.51 [0.13]	-0.17 [0.08]*
STG -> MTG	-0.02 [0.01]	7.93 [2.04]	0.53 [0.13]	-0.20 [0.09]*
STG -> FFG	0.01 [0.00]	-1.78 [4.72]	0.35 [0.14]	-0.02 [0.04]
STG -> IFG	-0.00 [0.00]	-3.76 [5.02]	0.32 [0.14]	0.02 [0.03]
MTG -> SMG	0.00 [0.01]	1.75 [4.37]	0.33 [0.14]	0.01 [0.02]
MTG -> IPL	-0.02 [0.01]	8.48 [2.00]	0.51 [0.12]	-0.17 [0.08]*
MTG -> ANG	0.00 [0.00]	0.54 [4.76]	0.34 [0.13]	0.00 [0.01]
MTG -> STG	-0.03 [0.01]	7.09 [2.23]	0.52 [0.14]	-0.18 [0.08]*
MTG -> FFG	-0.03 [0.01]	10.22 [1.96]	0.59 [0.12]	-0.26 [0.09]*
MTG -> IFG	0.00 [0.00]	-3.41 [4.71]	0.35 [0.14]	-0.01 [0.02]
FFG -> SMG	-0.00 [0.00]	6.75 [4.71]	0.35 [0.13]	-0.02 [0.03]
FFG -> IPL	-0.00 [0.00]	3.30 [5.07]	0.33 [0.14]	0.01 [0.02]
FFG -> ANG	-0.02 [0.01]	7.93 [2.02]	0.47 [0.12]	-0.13 [0.07]
FFG -> STG	-0.01 [0.00]	1.09 [4.58]	0.34 [0.14]	-0.01 [0.02]
FFG -> MTG	-0.03 [0.01]	8.42 [1.97]	0.55 [0.13]	-0.21 [0.08]*
FFG -> IFG	-0.01 [0.01]	10.71 [3.19]	0.44 [0.13]	-0.11 [0.07]
IFG -> SMG	-0.00 [0.00]	0.90 [5.16]	0.35 [0.15]	-0.01 [0.05]
IFG -> IPL	-0.03 [0.01]	6.13 [2.15]	0.49 [0.14]	-0.15 [0.07]*
IFG -> ANG	0.01 [0.00]	-3.83 [5.65]	0.36 [0.14]	-0.03 [0.04]
IFG -> STG	-0.01 [0.01]	6.87 [2.43]	0.43 [0.13]	-0.09 [0.06]
IFG -> MTG	0.00 [0.00]	-8.39 [4.68]	0.37 [0.13]	-0.03 [0.04]
IFG -> FFG	-0.02 [0.01]	8.19 [2.18]	0.52 [0.13]	-0.18 [0.08]*

Supplementary table 12: The table shows all effects (direct and indirect) of mediation analysis for developmental factor (PC3) as predictors, temporal directed partial coherence as a measure for directed functional connectivity as mediators and reading comprehension (RCOMP) subtest of Reading Inventory and Scholastic Evaluation (RISE) as the output measures. The values presented are regression estimates (unstandardized beta values) and standard error. All significant ($p < 0.05$, corrected using BH procedure) indirect effects are highlighted with the red font and asterisks and the corresponding significant direct effects are highlighted with red font.

Correlations (PC1)	Age_at_HA_fit	Age_first_CI	Deafness_prior_left_CI	Deafness_prior_first_CI	WRDC	VOC	MORPH	SEN	EFFIC	RCOMP
Age_at_HA_fit	1	.665**	.536**	.665**	-.388**	-0.264	-.315*	-.371**	-0.209	-.290*
Age_first_CI		1	.803**	1.000**	-0.269	-0.143	-0.137	-0.17	-0.09	-0.132
Deafness_prior_left_CI			1	.803**	-0.172	-0.055	-0.038	-0.103	0.013	-0.039
Deafness_prior_first_CI				1	-0.269	-0.143	-0.137	-0.17	-0.09	-0.132
WRDC_SS					1	.849**	.846**	.815**	.726**	.716**
VOC_SS						1	.893**	.770**	.788**	.831**
MORPH_SS							1	.827**	.786**	.830**
SEN_SS								1	.721**	.731**
EFFIC_SS									1	.840**
RCOMP_SS										1

Correlations (PC3)	WRDC	VOC	MORPH	SEN	EFFIC	RCOMP	Age
Age	0.145	.304*	.328*	0.125	.388**	.339*	1
HeadSize	0.108	.330*	0.254	0.049	0.237	.306*	.505**

Supplementary table 13: Correlations between clinical measures within each principal component and reading measures.
